# Effect of valve lesion on venous valve cycle: A modified immersed finite element modeling

**DOI:** 10.1371/journal.pone.0213012

**Published:** 2019-03-04

**Authors:** Xiang Liu, Lisheng Liu

**Affiliations:** 1 School of Science, Wuhan University of Technology, Wuhan, China; 2 Hubei Key Laboratory of Theory and Application of Advanced Materials Mechanics, Wuhan University of Technology, Wuhan, China; 3 State Key Laboratory of Advanced Technology of Materials Synthesis and Processing, Wuhan University of Technology, Wuhan, China; Politecnico di Milano, ITALY

## Abstract

The present study aimed to understand the effect of venous valve lesion on the valve cycle. A modified immersed finite element method was used to model the blood–tissue interactions in the pathological vein. The contact process between leaflets or between leaflet and sinus was evaluated using an adhesive contact method. The venous valve modeling was validated by comparing the results of the healthy valve with those of experiments and other simulations. Four valve lesions induced by the abnormal elasticity variation were considered for the unhealthy valve: fibrosis, atrophy, incomplete fibrosis, and incomplete atrophy. The opening orifice area was inversely proportional to the structural stiffness of the valve, while the transvalvular flow velocity was proportional to the structural stiffness of the valve. The stiffening of the fibrotic leaflet led to a decrease in the orifice area and a stronger jet. The leaflet and blood wall shear stress (WSS) in fibrosis was the highest. The softening of the atrophic leaflet resulted in overly soft behavior. The venous incompetence and reflux were observed in atrophy. Also, the atrophic leaflet in incomplete atrophy exhibited weak resistance to the hemodynamic action, and the valve was reluctant to be closed owing to the large rotation of the healthy leaflet. Low blood WSS and maximum leaflet WSS existed in all the cases. A less biologically favorable condition was found especially in the fibrotic leaflet, involving a higher mechanical cost. This study provided an insight into the venous valve lesion, which might help understand the valve mechanism of the diseased vein. These findings will be more useful when the biology is also understood. Thus, more biological studies are needed.

## Introduction

Chronic venous disease (CVI) is common in Western countries. In Europe, the cost to society owing to this disease has already exceeded 10 million Euros per million inhabitants per year [1,2]. In the USA, the varicose veins have afflicted up to 35% of adults [3]. Currently, the awareness of valve dysfunction, which contributes to varicose veins and venous phlebitis, has increased [4]. It is reported that [5,6] the valve dysfunction is mostly induced by valve failure and insufficiency. The prosthetic valve is a primary solution to treat the insufficient valve because the surgical correction is still technically challenging. A basic understanding of venous dynamics and valve functions, especially related to the diseased valve, is required to guide the design and implantation of valve prosthesis.

The development of inspection techniques [7–9] facilitated the knowledge of the morphology and functions of the healthy and insufficient valves. The typical valve lesions, their pathological behaviors, and their distinct features relative to the healthy ones have been observed. The *ex vivo* experiments [10] provided a means to mimic the valve mechanics and hemodynamics in the diseased vein. Certain valve damages, such as slit and rupture in artificial valves, could lead to a basic understanding of tailor-made reflux and leakage. Nevertheless, the actual physical mechanism of the diseased valves still remains unclear [11]. Especially the pathology is complicated and occurs irregularly, and the measurement of the mechanotransduction during the blood–valve interaction is limited. Thus, it is still challenging *in vivo*/*in vitro* to understand the valve dynamics and hemodynamics in the diseased vein clearly.

Computational modeling is a convenient tool to simulate the physiological valve and obtain the corresponding quantities [12–15]. Buxton et al. [12] employed a spring lattice model to illustrate the basic physics of vein valves. They investigated the dynamics of the valve opening area, and captured the unidirectional nature of the blood flow across the venous valve. Owing to a few reports on the mechanical properties [16,17], limited studies have explored venous valve modeling, particularly for the pathological cases [18–22] with insufficient biological knowledge [4]. Of the existing numerical studies, only Soifer et al. [19] studied the effects of stiffened venous valves on the neighboring valve using the arbitrary Lagrange–Eulerian (ALE) method. Simão et al. [18] and Ariane et al. [20,21] modeled the interaction between the agglomeration and the vein and studied the clotting dynamics and its effect on the reverse flow. Chen et al. [22] studied the helical flow induced by the relative positions of the valves and its corresponding effects on the stagnation. These studies are essential steps in studying the pathological vein valve, and they provided useful information on the hemodynamics around the valve and improved the relevant understandings. Nevertheless, studies related to the valve lesions induced by the abnormal elasticity are inadequate, especially when the abnormal elastic property is reported as one significant etiology of valve disease [2,6].

Inspired by the aforementioned issues, a modified immersed finite element method (IFEM) [23] was adopted in this study to investigate the effect of valve lesions, namely, fibrotic and atrophic remodeling of the valves [24,25]. The employment of IFEM could consider finite deformation of the venous tissues immersed in the background fluid, without high computational cost and complicated techniques for re-meshing on the fluid–solid interface. The modifications on the original IFEM, ghost fluid [26], and adhesive contact could approximate the physical interaction between the blood and the tissue, or between the tissues. Successful applications of IFEM to aortic valve modeling [27] and of the adhesive contact method to cell–matrix contact [28] have demonstrated their feasibilities in this study. In addition, studies on heart and aortic valve modeling [29,30] have revealed that pressure and velocity fields of three-dimensional (3D) and two-dimensional (2D) models are comparable. A 2D finite element modeling was adopted in this study for computational efficiency. With the use of this finite element modeling, a benchmark problem of normal valve modeling could be verified by comparing the results with the existing data. The resulting geometric orifice area (GOA) [31], volumetric flow rate [32], wall shear stress (WSS) [19], and mechanical cost function [33] in pathological cases were further compared between the healthy and the unhealthy valves to understand the effect of valve lesions on the valve dynamics and venous hemodynamics.

This study comprises the following sections. Section 2 introduces the employed numerical algorithms and computational technique. Section 3 describes the finite element modeling along with parameters of the healthy and pathological veins. Section 4 presents and compares computational results of the veins. Section 5 summarizes the results, followed by the discussion. Section 6 presents the relevant conclusion.

## Numerical method

The key ingredients of the numerical method were the modified immersed finite element fluid solver, hyperelastic structural solver, adhesive contact algorithm, and fluid–structure coupling approach. The nomenclature used in this study is listed in Table 1.

**Table 1:** List of principal quantities.

Quantities	Symbol	Units
**Spatial coordinate**	***x*, *x***^*s*^	cm
**Material coordinate**	***X***^*s*^	cm
**Velocity**	***v*, *v***^*f*^**, *v***^*s*^	cm/s
**Displacement**	***d***^*s*^	cm
**Density**	*ρ*, *ρ*^*f*^, *ρ*^*s*^	g/cm^3^
**Dynamic viscosity**	*μ*, *μ*^*f*^	g/(cm·s)
**Damping coefficient**	*c*	g/s
**Force**	***F***	kg·m/s^2^
**Body force**	***f***	m/s^2^
**Cauchy stress**	***σ***^*f*^**, *σ***^*s*^	g/(cm·s^2^)
**Time**	*T*	s

### Modified immersed finite element fluid solver

**Fig 1:**
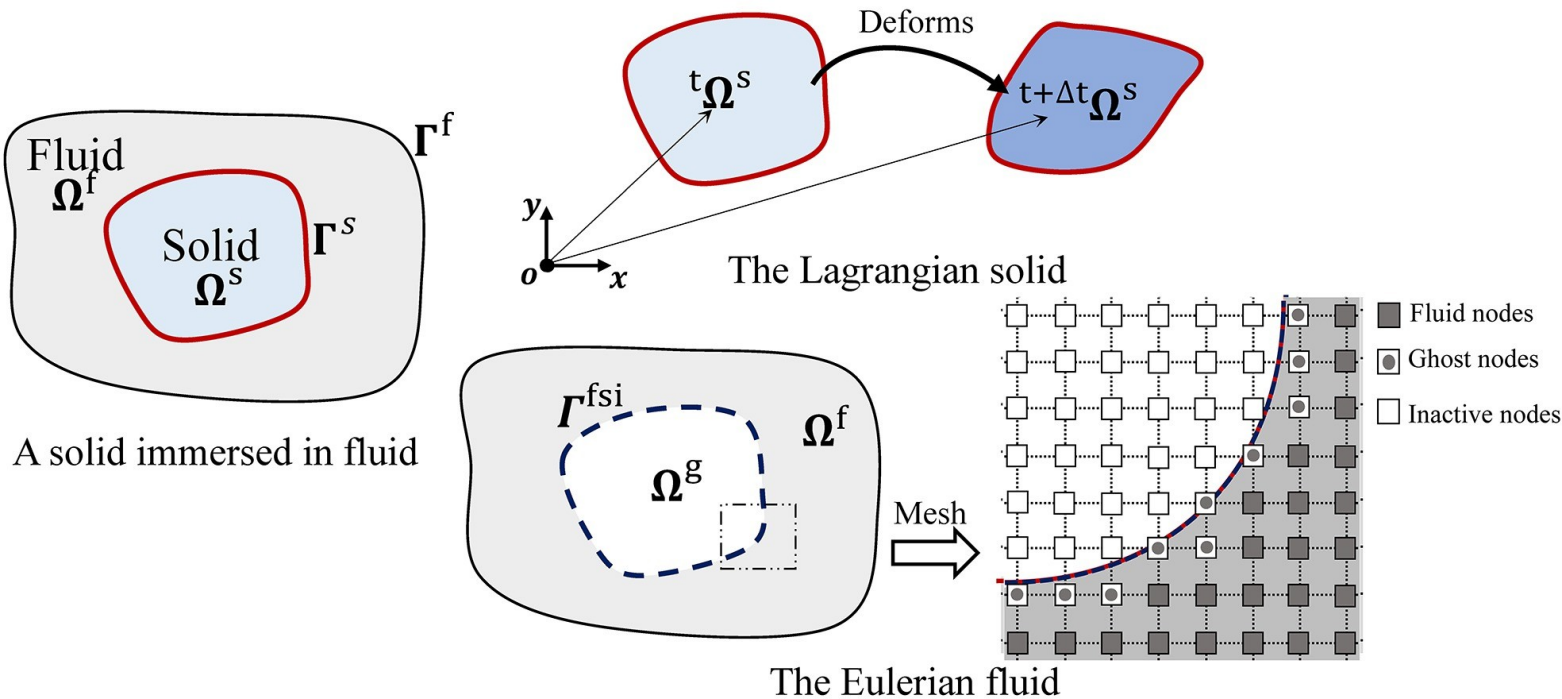
Computational domain decomposition.

Based on the original IFEM [23], a Lagrangian solid *Ω*^*s*^ fully immersed in a Eulerian fluid *Ω* was used. The solid mesh and fluid mesh were independent so that no body-fitted mesh or re-meshing costs were involved in the computation. Moreover, the modified IFEM introduced an idea of the ghost fluid [26] by replacing the previous artificial fluid [23] with the ghost fluid. Then, the Eulerian fluid domain *Ω* consisted of a real fluid domain *Ω*^*f*^ and ghost fluid domain *Ω*^*g*^, as shown in Fig 1. The ghost fluid domain *Ω*^*g*^ included both ghost and inactive nodes. The property parameters of the ghost fluid were infinitesimal, and the inactive nodes were not counted into the computations of the Navier–Stokes (N–S) equations and the fluid–structure interaction (FSI). Using a Dirac delta function *δ*(***x***), density and dynamic viscosity of the Eulerian fluid domain *Ω* were calculated as follows:

$$\rho =\int_{\Omega }\rho^{f}\delta (x-x^{s})d\Omega ;\;\mu =\int_{\Omega }\mu^{f}\delta (x-x^{s})d\Omega$$ (1)

with

$$\int_{\Omega }\delta (x-x^{s})d\Omega =\left\{ \begin{array}{c} 1,\;x\in \Omega^{f}\\ 0,\;x\in \Omega^{g} \end{array}\right.$$ (2)

where *ρ*^*f*^ is the fluid density and *μ*^*f*^ is the dynamic viscosity.

The governing equations were the unsteady and incompressible N–S equations and written as follows:

$$\{\begin{array}{l} v_{i,i}=0,\\ \rho \left[v_{i,t}+\left(v_{i,j}\right)v_{j}\right]=\sigma_{ij,j}+\rho f_{i}, \end{array}\;\mathrm{on}\;\Omega \times \left[0,T\right]$$ (3)

with

$$\sigma_{ij,j}=-p_{,i}+\mu (v_{i,j}+v_{j,i})\;on\;\Omega \times [0,T].$$ (4)

where *v*_*i*_ and *f*_*i*_ are the velocity and body force components in the *i*th direction, respectively; *σ*_*ij*_ is the quantity of the Cauchy stress tensor; and *p* is the pressure. A second-order fractional step finite element method [34] was used to improve the pressure stability in the N–S equations (see S1 Appendix).

After the introduction of the ghost fluid, an immersed sharp interface *Γ*^*isi*^ existed between *Ω*^*f*^ and *Ω*^*g*^. The velocity boundary conditions of the interface *Γ*^*isi*^ were exactly prescribed through the interpolation between *Γ*^*isi*^ and the solid boundary *Γ*^*s*^. Along with other prescribed conditions on *Γ*^*f*^, both the Dirichlet boundary condition *Γ*^*v*^ and the Neumann boundary condition *Γ*^*t*^ on the fluid domain *Ω* were employed:

$$\left\{ \begin{array}{l} {\left(v_{i}\right)}_{\Gamma^{v}}={\bar{u}}_{i},\;on\;\Gamma^{v}\in \Gamma^{v,f}\cup \Gamma^{v,isi};\\ {\left(\sigma_{ij}∙n_{j}\right)}_{\Gamma^{t}}={\bar{t}}_{i},\;on\;\Gamma^{t}\in \Gamma^{t,f}\cup \Gamma^{t,isi}. \end{array}\right.$$ (5)

where ${\bar{u}}_{i}$ and ${\bar{t}}_{i}$ are the prescribed velocity and traction, respectively; and *n*_*j*_ is the normal vector of *Γ*^*f*^ or *Γ*^*isi*^.

The Streamline-Upwind Petrov-Galerkin (SUPG) and Pressure-Stabilizing Petrov-Galerkin (PSPG) formulations were employed to solve the N–S equations [23]. The *Q1Q1* element of second-order accuracy was chosen for equal-order pairs of velocity and pressure. Employing the GMRES iterative algorithm [23,35], the discretized residuals of velocity $r_{Ii}^{v}$ and pressure $r_{I}^{p}$ were as follows:

$$\left\{ \begin{array}{l} r_{Ii}^{v}=\int_{\Omega }\left\{\tau^{lsic}\rho N_{I,i}v_{j,j}+(N_{I,i}+\tau^{supg}v_{k}N_{I,k})[\rho (v_{i,t}+v_{i,j}v_{i,j})-\sigma_{ij,j}-f_{i}]\right\}d\Omega \\ r_{I}^{p}=\int_{\Omega }\left\{\tau^{pspg}N_{I,i}[\rho (v_{i,t}+v_{i,j}v_{i,j})-\sigma_{ij,j}-\rho f_{i}]+N_{I}v_{j,j}\right\}d\Omega \end{array}\right.$$ (6)

were computed based on the Jacobian-free Newton–Raphson technique [36]. Here, *τ*^*supg*^, *τ*^*pspg*^, and *τ*^*lsic*^ denote the stabilized parameters in the SUPG and PSPG. *N*_*I*,*i*_ is the derivative of the shape function *N*_*I*_ (at node *I*) with respect to *x*_*i*_. For detailed derivations, one can refer to the study by Zhang et al. [23].

### Hyperelastic structural solver

Polynomial nonlinear functions were used for the hyperelastic model. Two types of constitutive laws were used to explain the behavior of the tissues [37]:

$$W^{wall}=c_{1}(\alpha -1)+c_{2}{\left(\alpha -1\right)}^{3}+c_{3}({Ι}_{1}-3)+c_{4}({Ι}_{1}-3)(\alpha -1){+c}_{5}{\left({Ι}_{1}-3\right)}^{2}$$ (7)

$$W^{valve}=c_{0}(\exp\left(c_{1}{\left({Ι}_{1}-3\right)}^{2}+c_{2}{\left(\alpha -1\right)}^{4}\right)-1)$$ (8)

where the five-parameter polynomial-type strain energy descriptor $W^{wall}$ is given for the wall tissue and the three-parameter exponential-type function $W^{valve}$ is given for the valve tissue; *c*_*i*_ is the material coefficient; and *I*_1_ and *α*^2^ are the stretch invariants in the “1” direction and fiber angle direction, respectively.

The general expression [38,39] for the Cauchy stress ***σ***^***s***^ in this type of material is written as follows:

$$\sigma_{ij}^{s}=-p^{s}+2\frac{\partial W}{\partial I_{1}}B_{ij}+\frac{\partial W}{\partial \alpha }F_{iL}N_{L}N_{M}F_{jM}$$ (9)

where *p*^*s*^ is a Lagrange multiplier enforcing incompressibility; ***F*** = ∂***x***^*s*^/∂***X***^*s*^ is the deformation gradient tensor; ***B*** = ***FF***^T^ is the left Cauchy–Green deformation tensor; and ***N***(***X***^*s*^) is the unit vector of the fiber-axis direction in the initial configuration.

The governing equation of solid dynamics is written as follows:

$$\sigma_{ij,j}^{s}+\rho^{s}f_{i}-\rho^{s}v_{i,t}^{s}-cv_{i}^{s}=0\;on\;\Omega^{s}\times [0,T].$$ (10)

where ***f*** is the body force, *ρ*^*s*^ is the density, ***v***^***s***^ is the velocity, and *c* is the damping coefficient. Using the Galerkin method, the dynamic (Eq 10) was transformed into the discretized formulation as follows:

$$M_{IJ}{\left(d_{Ii,t}^{s}\right)}_{,t}+C_{IJ}d_{Ii,t}^{s}+K_{IJ}d_{Ii}^{s}=F_{Ii}$$ (11)

where the mass matrix $M_{IJ}=\int_{\Omega^{s}}N_{I}\rho^{s}N_{J}d\Omega^{s}$, the stiffness matrix $K_{IJ}=\int_{\Omega^{s}}D_{ijkl}B_{Iij}B_{Jkl}d\Omega^{s}$, and the damping matrix *C*_*IJ*_ = *f*_*m*_*M*_*IJ*_ + *f*_*k*_*K*_*IJ*_, with *f*_*m*_ and *f*_*k*_ denoting the mass coefficient and the stiffness coefficient, respectively. The nodal force *F*_*Ii*_ was combined by the nodal traction $F_{Ii}^{t}=\int_{\Gamma^{s}}\sigma_{ij}^{s}N_{Jj}d\Gamma^{s}$ and the nodal body force $F_{Ii}^{b}=\int_{\Omega^{s}}\rho^{s}f_{i}^{s}N_{Ii}d\Omega^{s}$. The displacement of a solid element $d_{i}^{s}$ was discretized with the following interior nodes:

$$d_{i}^{s}=\sum_{I=1}^{nen}N_{I}d_{Ii}^{s}$$ (12)

where *nen* is the number of nodes per element. The integration was simplified through the use of isoparametric coordinates. Following this, the semi-discrete (Eq 11) was further discretized in time using the Newmark scheme [40]. The discrete equation is written as follows:

$$\left[\left(f_{m}+\frac{1}{\theta \Delta t}\right)M+\left(f_{k}+\theta \Delta t\right)K\right]d^{s,t}=\theta \Delta tF^{t}+\left(1-\theta \right)\Delta tF^{t-\Delta t}+\frac{1}{\theta }Md_{,t}^{s,t-\Delta t}+\left(f_{m}+\frac{1}{\theta \Delta t}\right)Md^{s,t-\Delta t}+\left[f_{k}-\left(1-\theta \right)\Delta t\right]Kd^{s,t-\Delta t}$$ (13)

where *θ* is a constant equaling to 0.5, *f*_*m*_ = 0.05, and *f*_*k*_ = 0.272. The matrices (in Eq 16) were formed by Cholesky factorization and assembled in “skyline” form [40]. The Cholesky forward and back-substitution [40] were employed to solve the equation systems.

### Adhesive contact between solids

**Fig 2:**
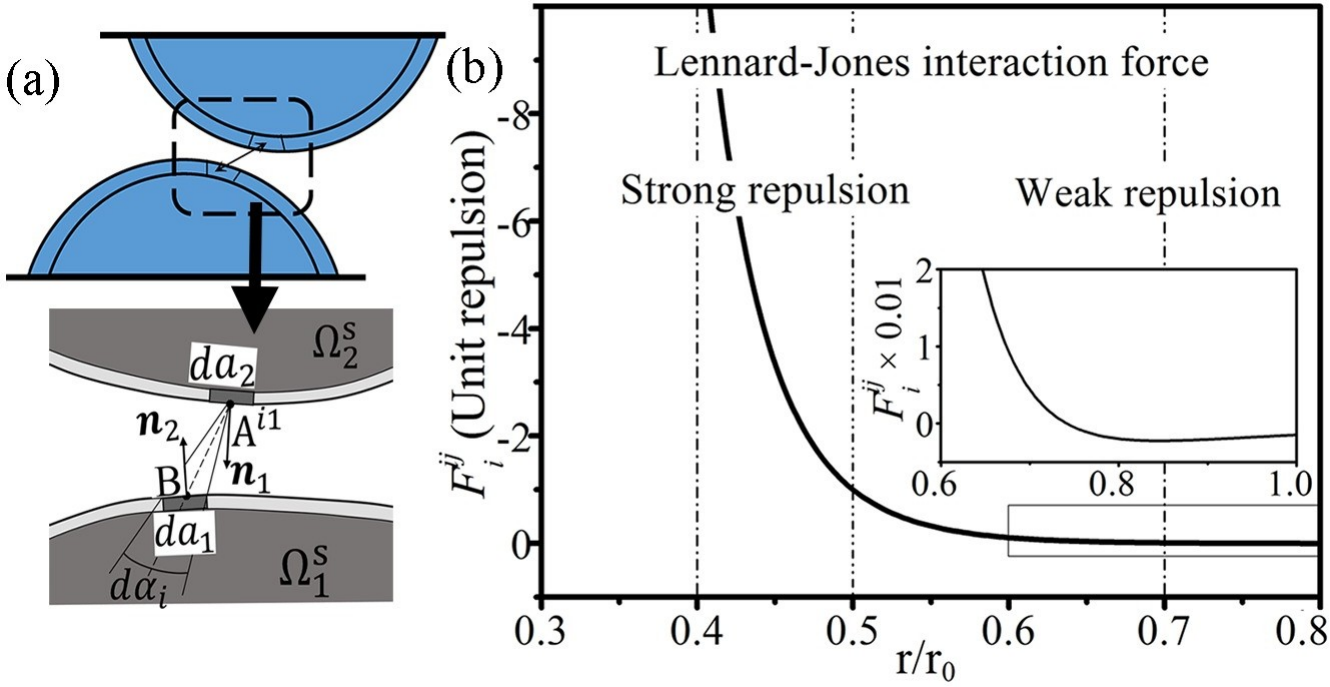
Schema of contact model. (a) The interaction force in the adhesive contact model. (b) The relationship between the contact force $F_{i}^{ij}$ and the distance *r*_0_.

Contact between tissues is a common phenomenon during venous valve cycle [8], such as valve closure. The adhesive contact algorithm [41] is used to build the contact model. As shown in Fig 2A, $\Gamma_{1,i}^{s}$ represents the *i*th infinitesimal surface of the solid $\Omega_{1}^{s}$, and $\Gamma_{1,j}^{s}$ represents the *j*th infinitesimal surface of the solid $\Omega_{2}^{s}$. The contact force $F_{i}^{ij}$ between them was a long-range force associated with an inter-body interaction potential *ϕ*(*r*) [41] as follows:

$$F_{i}^{ij}=-\beta_{i}\beta_{j}n_{j}da_{j}d\alpha_{}\;\int_{s_{ij}}^{\infty }\phi (r)r\;dr$$ (14)

where *β*_*j*_, *da*_*j*_, and ***n***_*j*_ are the dimensionless density, the area, and the normal vector of $\Gamma_{1,j}^{s}$, respectively; *β*_*i*_ and *da*_*i*_ are the variables of $\Gamma_{1,i}^{s}$; *dα* is the angle of the cone formed by $\Gamma_{1,j}^{s}$ and the mid-point *B* of $\Gamma_{1,i}^{s}$; and *s*_*ij*_ is the distance from point *B* to $\Gamma_{1,j}^{s}$. A 12–6 Lennard-Jones model was chosen for the potential:

$$\phi (r)=\varepsilon \left[{\left(\frac{r_{0}}{r}\right)}^{12}-2{\left(\frac{r_{0}}{r}\right)}^{6}\right]$$ (15)

where *ε* is the potential well, *r*_0_ is the equilibrium distance, *r* is the distance variable, and *r* ∈ [*s*_*ij*_, ∞]. Thus, counting the force over all the contact areas creates a strong repulsion to enforce the nonpenetrability condition between two tissues. With different values of *ε*, the contact condition could be soft or hard according to the situation, while additional attention was paid to the time step size.

As illustrated in Fig 2B, a dimensionless distance $\frac{r}{r_{0}}$ was used to help understand the quantitative relationship between *r* and repulsion. When $\frac{r}{r_{0}}\in [0.4,0.7]$, it was flexible to adjust the contact force so that the potential penetration was opposed timely and the solids were in suitable positions. For the efficiency, the contact computation could only consider the elements adjacent to the boundaries of the leaflets and the sinus. In the existing valve studies [31,39], the structure and movement of the bileaflet valves were nearly symmetric to the longitudinal cross-section of a vessel. Thus, contact between venous tissues also occurred at the central transverse plane.

### Fluid–structure coupling

The fluid–structure coupling was achieved through the interpolation between the immersed sharp interface *Γ*^*isi*^ and the solid boundary *Γ*^*s*^. Representative elements adjacent to *Γ*^*isi*^ and *Γ*^*s*^ were chosen to specify the interpolations.

**Fig 3:**
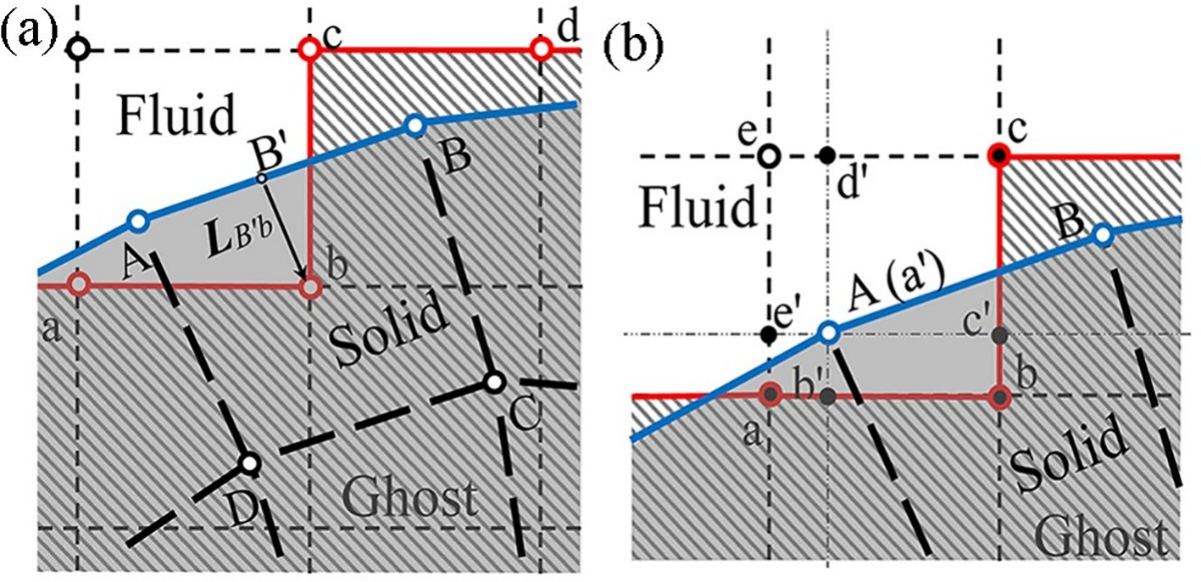
Schema of fluid–structure interaction models. (a) The interpolation of velocity from solid to fluid. (b) The interpolation of traction from fluid to solid.

As illustrated in Fig 3A, interpolation of velocity from *Γ*^*s*^ to *Γ*^*isi*^ was implemented by transmitting velocity $v_{B}^{s}$ to fluid node *b* as follows:

$$v_{b}=v_{B^{\prime }}^{s}+\nabla v_{B^{\prime }}^{s}l_{B\prime b}$$ (16)

where ∇ denotes the gradient operator with respect to ***x*** and $l_{B\prime b}=x_{b}-x_{B^{\prime }}^{s}$. Node *B'* is a node shared by the fluid and solid so that $v_{B^{\prime }}^{s}=v^{s}(X_{B^{\prime }}^{s})=v_{B\prime }=v(x_{B^{\prime }})$.

The fluid stress upon *Γ*^*s*^ was transmitted from *Γ*^*isi*^ using the bilinear feature of the shape function, which neglected the shear stress due to the no-slip condition, as shown in Fig 3B. Several auxiliary nodes *b'*, *c'*, *d'*, and *e'* were assumed on the boundaries of the fluid element. Fluid node *a'* (or the solid node A) was calculated through bilinear interpolations as follows:

$$\left\{ \begin{array}{l} p_{a\prime }=\alpha \beta p_{a}+\beta (1-\alpha )p_{b}+(1-\alpha )(1-\beta )p_{c}+\alpha (1-\beta )p_{e}\\ v_{a\prime }=\alpha \beta v_{a}+\beta (1-\alpha )v_{b}+(1-\alpha )(1-\beta )v_{c}+\alpha (1-\beta )v_{e} \end{array}\right.$$ (17)

with *α* = *l*_*ed*′_/*l*_*ec*_ = *l*_*ab*′_/*l*_*ab*_ and *β* = *l*_*bc*′_/*l*_*bc*_ = *l*_*ae*′_/*l*_*ae*_, *l*_*ij*_ denoting the distance between nodes *i* and *j*. Afterward, the fluid traction ***t***^*f*^ upon the solid was calculated as ***t***^*s*^ = −***t***^*f*^ according to Newton’s third law. A mesh size ratio of fluid to solid should be between 0.5 and 2.0 to facilitate the interpolations [23], which was 1.0 in this study. The sensitivity analysis of mesh size has been analyzed and properly chosen for the fluid-structure coupling, along with that of the time step (see S2A Appendix and S1 Fig).

### Numerical implementation

The proposed FSI method included a fluid module, a solid module, a contact module, and two interpolation modules. By employing an explicit coupling scheme, the algorithm procedure was as follows:

1. Solid governing equations were solved with the previous hydrodynamic force and contact force; coordinates of the deformed solid were updated.
2. The density and dynamic viscosity of the entire computational domain were updated, and the velocities of the solid were interpolated onto the immersed interface within the domain.
3. The N–S equations on the fluid domain were solved with the prescribed conditions at boundaries and the interface.
4. The hydrodynamic forces on the solid were then calculated at the solid boundary via an interpolation scheme on the flow grid.
5. The existence of contact was predicted according to the distance between solids. The contact force was calculated if it existed, then moving to the next step.

As mentioned earlier, the explicit coupling was quite simple, robust, and efficient. The implicit coupling could be easily implemented, if needed, by iterating between the fluid and solid solvers at each time step. Finally, the FSI method, where the modified IFEM and adhesive contact algorithm were employed, was implemented in Fortran 90.

## Finite element modeling of vein

### Geometry model

**Fig 4:**
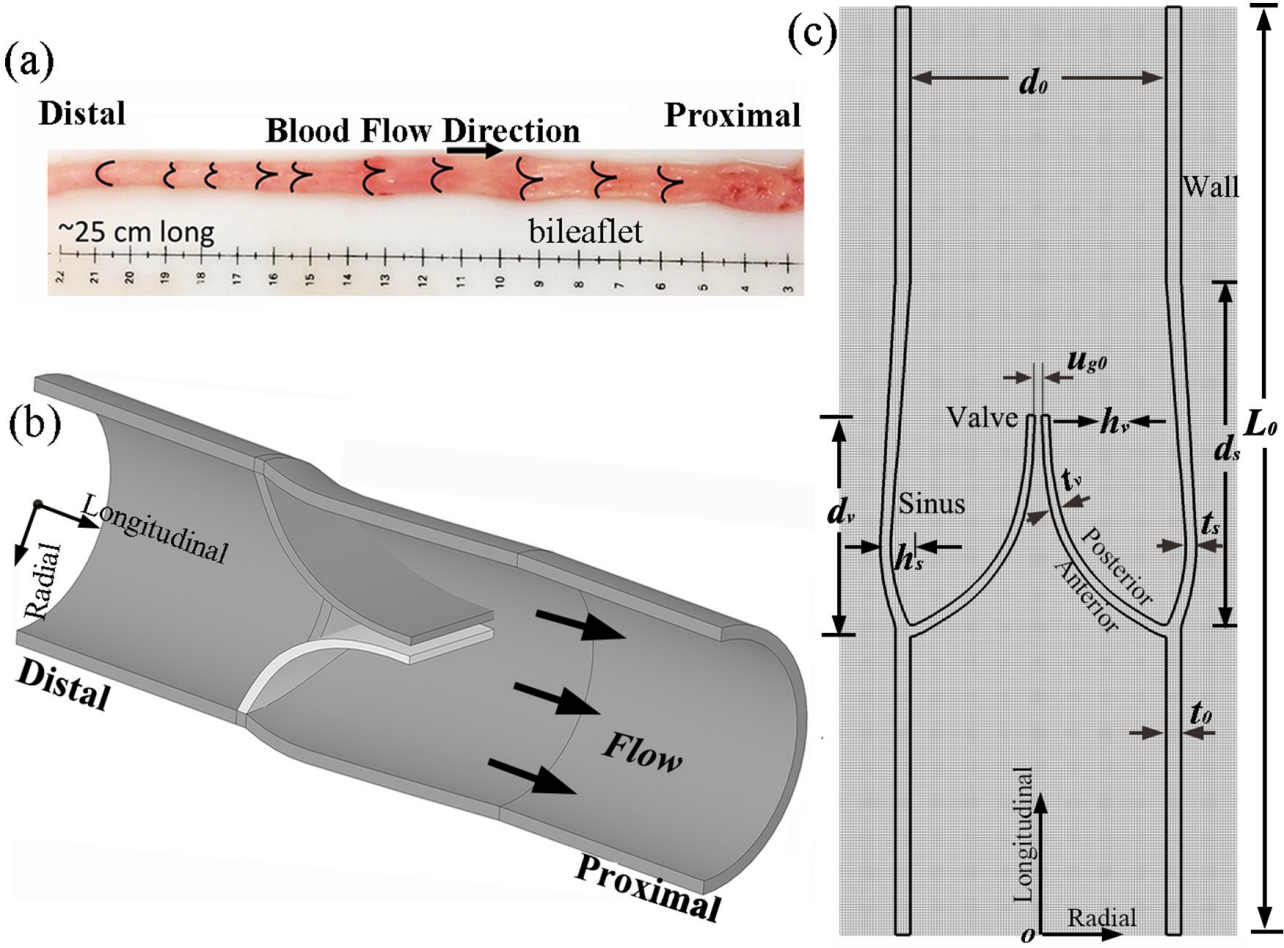
Geometry and size of the venous model. (a) Vein sample (obtained from [17]). (b) 3D structure. (c) 2D model.

The finite element model was constructed according to the characteristic dimensions of a bovine saphenous vein to ensure consistent dimensions in a vein structure [17], as illustrated in Fig 4. The vein model was featured with a thin wall, a symmetrical bileaflet valve, and sinus pockets. The valve leaflet was semi-lunar shaped and proximally directed, set 0.8 cm away from the distal end of the vein. The detailed dimensional parameters are listed in Table 2. Since the sinus geometry was unclear, its bugled height *h*_*s*_ was set as 0.05 cm to ensure that the mean diameter of the vein was consistent with that in [17].

**Table 2:** Dimensional parameters of the vein model.

Structure	Dimension	Label	Direction	Size (cm)
**Wall**	Length	*L*_0_	Longitudinal	2.50
**Wall**	Luminal diameter	*d*_0_	Radial	0.691
**Wall**	Thickness	*t*_0_	Radial	0.040
**Valve**	Depth	*d*_*v*_	Longitudinal	0.573
**Valve**	Height	*h*_*v*_	Radial	0.310
**Valve**	Thickness	*t*_*v*_	Radial	0.020
**Valve**	Gap	*u*_*g0*_	Radial	0.018
**Sinus**	Depth	*d*_*s*_	Longitudinal	0.922
**Sinus**	Bulged height	*h*_*s*_	Radial	0.050
**Sinus**	Thickness	*t*_*v*_	Radial	0.030

According to the principle of IFEM [23,27], the vein was immersed in the Eulerian fluid domain of size 2.5 × 1.071 cm^2^. The distensions of the wall and the sinus were thus considered. Owing to the requirement in adhesive contact, an initial gap *u*_*g*0_ = 0.018 cm between the leaflets was preserved to prevent the initial penetration.

### Material properties

Recent histological studies [16,42] have indicated that the local nature of the venous valves is transversely isotropic in the longitudinal cross-section. This information helped plausibly assume that the tissues of the valve and wall were pseudoelastic and locally isotropic with respect to the fiber axis [16]. When the constitutive (Eqs 10 and 11) of the hyperelastic model were employed, the fiber-reinforced terms involving *α* were simplified in the transverse plane. The property parameters of the vein based on previous data [42,43] are given in Table 3.

**Table 3:** Material properties of the three parts of the vein model.

Part	Density (g/cm^3^)	C0 (kPa)	C1	C3 (kPa)	C5 (kPa)
**Wall** [10]	1.10	–	–	2.00	187.5
**Valve** [16,17]	1.10	417	0.06	–	–
**Sinus** [16,17]	1.10	–	–	0.50	46.875

Note that the parameters were not given for the corresponding tissue due to the fiber-reinforced terms. Similar to the existing numerical studies [12,19], the elasticity of the valve was specially adapted to allow the physiological valve behavior reported by Lurie et al. [8] and Mirnajafi et al. [44]. The characteristic parameters C0, C1, C3, and C5 were scaled. The initial elastic modulus of the wall and valve tissues was 1.5 MPa [10,19] and 200.0 kPa, respectively [16,17]. The mechanical property of venous sinus was assumed a quarter of that of the wall material owing to the scanty information available about it.

In the 2D fluid domain, blood was modeled as a Newtonian liquid. The density *ρ*^*f*^ of full hematocrit was 1.08 g/cm^3^ [45]. The dynamic viscosity *μ*^*f*^ was 0.0036 Pa∙s at 37 degree centigrade [46]. A plane strain assumption could be adopted for the 2D simulation because the length of the valve specimen was far greater than the thickness [16,17].

### Boundary conditions

**Fig 5:**
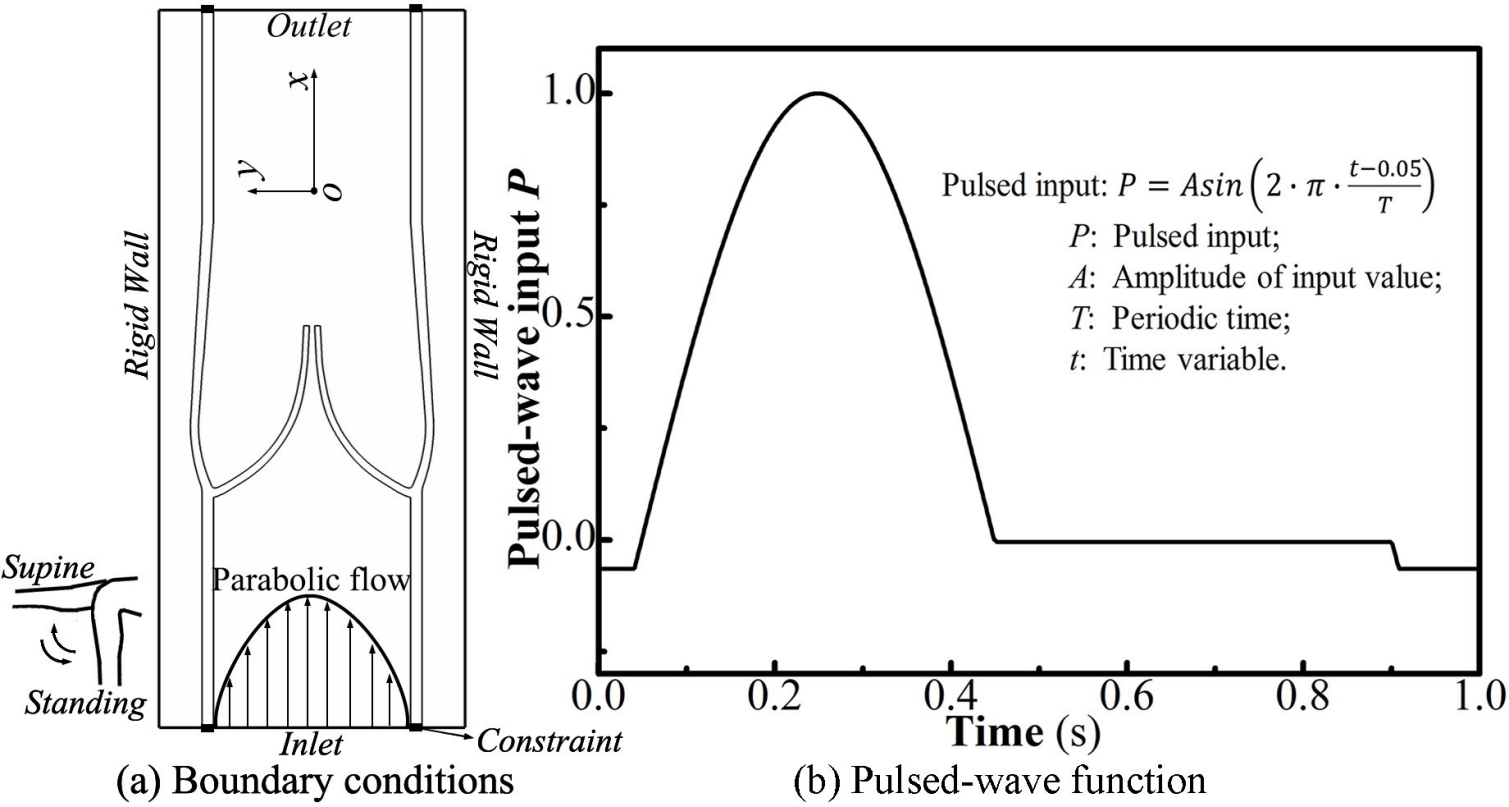
Prescribed boundary conditions. (a) Inlet pressure boundary condition, inlet velocity boundary condition, and gravity. (b) Pulsed-wave function.

For a normal valve cycle, the ongoing force of flow is strongly related to gravity and muscle pump [8]. Physiologists suggested that [4] hydrostatic pressures below the right atrium derived from the weight of the blood column and were expressed as a constant multiplier (0.77 mm Hg/cm) of the vertical distance in centimeters. Along with a muscular pump *p*_pump_, a maximum pressure gradient of 4.0 mm Hg (Δ*p* = *ρ*^*f*^*gL*_0_ + *p*_pump_) was then imposed for alternate positions of supine and standing, as shown in Fig 5. The inflow at the inlet was set as parabolic and pulsatile flow at 60 cycles/min, mimicking the venous pump. In each cycle, a short initial period of 0.05 s was set in the pulsed-wave function to relax the leaflets in an initial closed state. Except for the inlet and the outlet, other boundaries of the fluid domain had constraints in both the pressure and velocity. Finally, the vein was constrained at both ends of the wall.

### Finite element modeling

In the 2D simulation, either the mesh size or the time step size was based on the considerations of mesh sensitivity, time step sensitivity, Courant–Friedrichs–Lewy condition, and ghost node arrangement. With a mean mesh size of 0.008 cm, the vein structure consisted of 2864 quadrilateral elements: 1408 for the wall, 632 for the valve, and 824 for the sinus. The Eulerian fluid domain consisted of 47,762 quadrilateral elements. The time step size was 0.0004 s. Regarding the contact parameters, the equilibrium distance *σ*_0_ was 2*u*_*g*0_, namely, 0.036 cm; the potential well *ε* was –4.0 J to keep a weak repulsion at the equilibrium position; and the dimensionless densities *β*_*i*_ and *β*_*j*_ were 1.0 for the venous tissues.

### Typical valve lesions

Recent studies have estimated that the incidence as a cause of CVI was approximately 80% for post-thrombotic damage and 20% for primary valvular incompetence. In post-thrombotic syndrome, the residual thrombus is replaced by fibrous tissue and the fibrotic process is entrapped in valve leaflets [4]. According to the angioscopic evidence of valvular incompetence [7], 58.2% assessed cases are classified as depressed valve or atrophy. For the high prevalence, fibrosis and atrophy are two typical valve lesions investigated in this study.

Considering that the valve lesions are irregular in the biological tissues, the remodeling was assumed covering the total leaflet, including the alterations of elasticity and thickness. The fibrosclerotic remodeling was considered as thickening (25%) and damaged elasticity (stiffer) [47]. The elasticity of the atrophic leaflet was regarded as weakened [7]. For the incomplete lesion, one leaflet of the valve was assumed fibrotic or atrophic. Finally, four typical valve lesions are given in Table 4.

**Table 4:** Material properties of the bileaflet valves with mechanical lesion.

Case	Valve lesion	Leaflet	State	C0 (kPa)
^a^ **CFV**	Fibrosis	^b^ No. 1	Fibrotic	4170
		No. 2	Fibrotic	4170
**CAV**	Atrophy	No. 1	Atrophic	41.7
		No. 2	Atrophic	41.7
**IFV**	Incomplete fibrosis	No. 1	Fibrotic	4170
		No. 2	Healthy	417
**IAV**	Incomplete atrophy	No. 1	Atrophic	41.7
		No. 2	Healthy	417

^a^ CFV, completely fibrotic valve; CAV, completely atrophic valve; IFV, incompletely fibrotic valve; IAV, incompletely atrophic valve.

^b^ No. 1 and No. 2 represent the left and right leaflets illustrated in Fig 5A, respectively.

Similar to a recent study on pathological valves [19], the elasticity of the fibrotic leaflet increased 10 times. Following such a rule, the atrophic leaflet was modeled by reducing the modulus to 1/10. Additionally, the dimension and geometry of the pathological models were assumed the same as those in the normal vein, along with the same inlet flow conditions.

## Results

Two groups of simulations were reported in this study. The first was intended to validate the computed results of the normal valve both qualitatively and quantitatively against the physiological measurements and numerical simulations. The second focused on the effects of the valve lesions on the valve dynamics and venous hemodynamics. The results of different valves were compared to display the characteristics of valve lesions. For each case, only a valve cycle was presented, which took 25.07 h in the Intel Core i7-4790HQ processor with the main frequency 3.60 GHz.

### Validation of normal valve model

**Fig 6:**
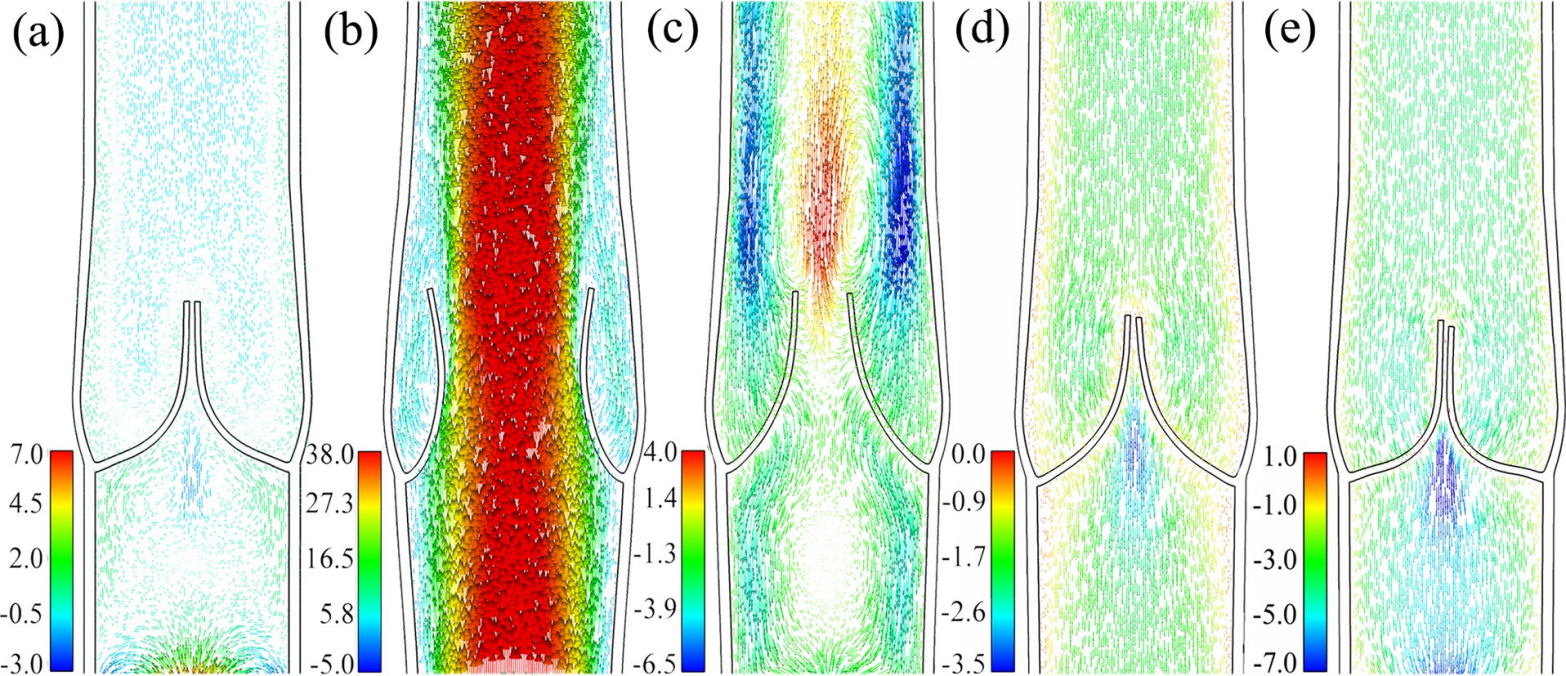
Various valve configurations and flow streamlines in a normal valve cycle. (a) In the opening phase, 0.08 s. (b) In the equilibrium phase, 0.30 s. (c) In the closing phase, 0.51 s. (d) In the closed phase, 0.8 s. (e) In the closed phase, 1.0 s.

A normal valve cycle in physiology includes four phases: opening, equilibrium, closing, and closed [8]. Fig 6 depicts various valve configurations and planar flow streamlines corresponding to each phase. In the opening phase (Fig 6A), rapid inflowing blood led to strong ejection on the leaflets. The venous tissues were pushed and distended owing to the transvalvular pressure gradient, and the leaflets rotated around the hinge point immediately and made an angle of nearly 30 degrees with the wall. Then, the ejection was separated from the leaflet surface and flowed across the orifice. In the equilibrium phase (Fig 6B), the leaflets oscillated and swung in the maximum opening position. In the closing phase (Fig 6C), the velocity of the ejection decreased, the venous tissues recoiled, and the blood reversely flowed from the distal outlet. The closing phase ended when the leaflets were in “contact” (Fig 6D). The reverse flow was prevented from flowing across the valve. In the closed phase, the flow pattern varied little while the coaption of the leaflets was becoming more evident with greater amounts of their surface contacting together. The valve performance and flow pattern complied with the reported physiological phenomena in the corresponding phase [8].

**Fig 7:**
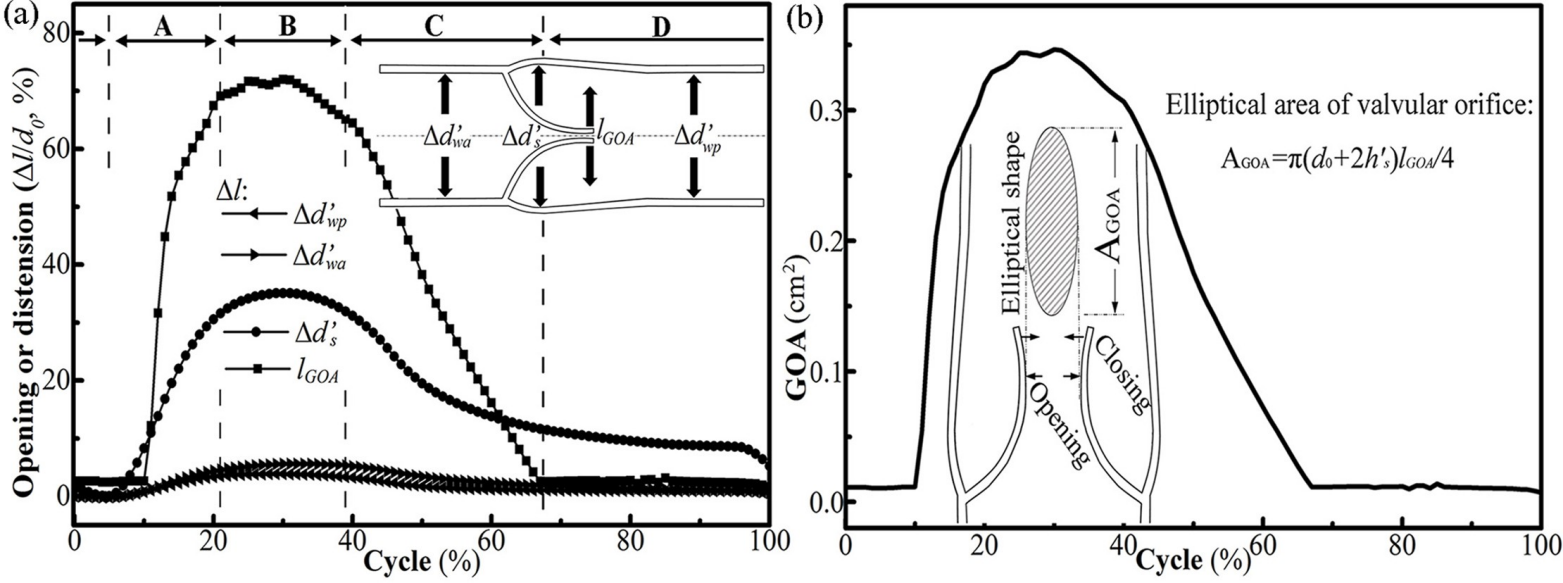
(a) Time variations of the venous behavior: A, opening phase, 0.05–0.20 s; B, equilibrium phase, 0.20–0.39 s; C, closing phase, 0.39–0.68 s; and D, closed phase, 0.68–1.05 s. Δ*d’*_*wa*_ is the displacement of the marked position on the anterior wall, Δ*d’*_*wp*_ is the displacement of the marked position on the posterior wall, and Δ*d’*_*s*_ is the displacement of the sinus. (b) Time variation of the geometric orifice area A_GOA_.

The duration of each phase could be further understood according to the time-varying behavior of the venous tissues, as shown in Fig 7. The maximum orifice size *l*_GOA_ (0.494 cm) appeared in the equilibrium phase, which was 71.4% of the initial luminal diameter *d*_*0*_. This opening amplitude was consistent with the physiological situation (60%–70%) in the saphenous veins [48,49]. Also, the distensions of the sinus and wall contributed to the opening of the orifice. Previous studies reported [4] that the valve had a funnel shape with an elliptic-shaped orifice when opening. Thus, the orifice area was calculated as $A_{GOA}=\pi (d_{0}+2h_{s}^{\prime })l_{GOA}/4$, with the long axis of *d*_*0*_ + 2*h’*_s_ and the short axis of *l*_GOA_ [8]. Time variation of A_GOA_ is given in Fig 7, and the law was the same as that of *l*_GOA_ after including the distension of venous sinus.

**Fig 8:**
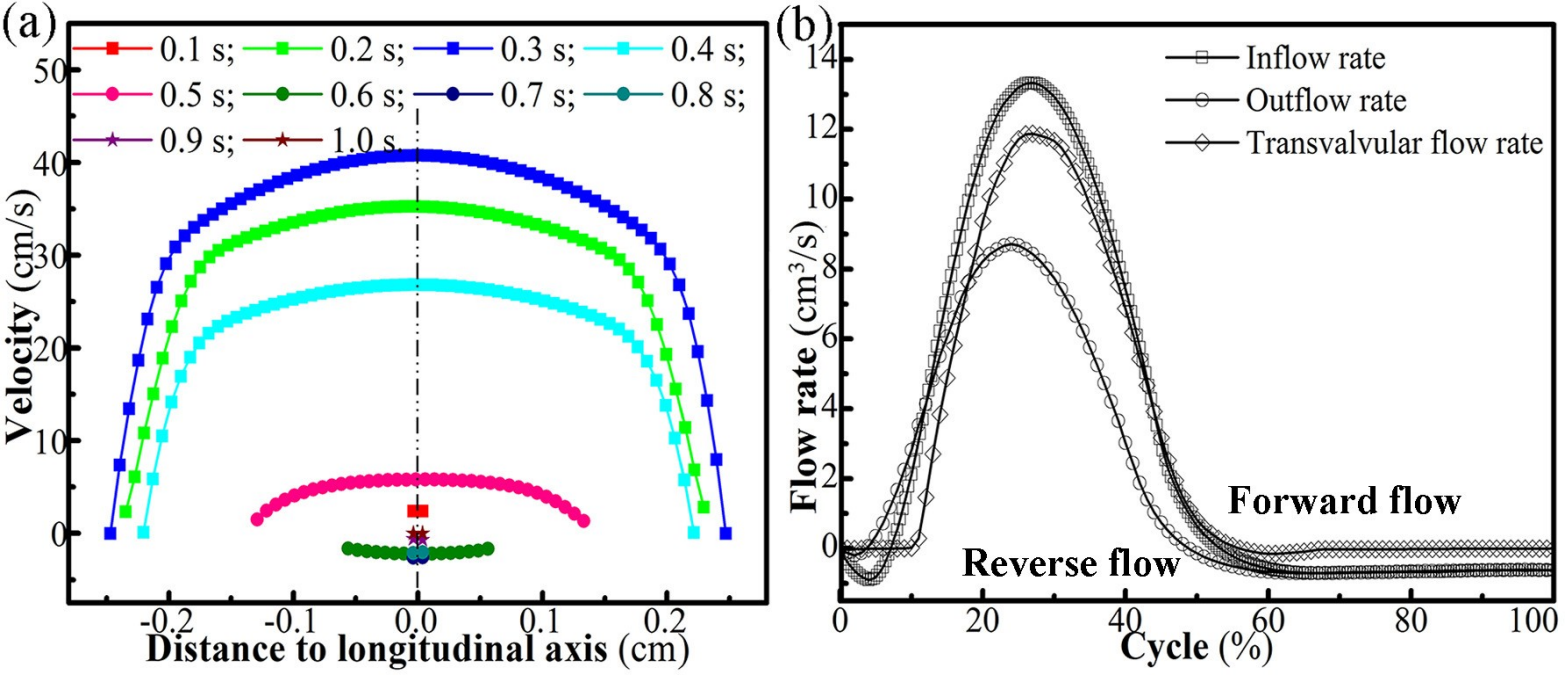
(a) Velocity distributions at the orifice. (b) Flow rate at the inlet, orifice, and outlet.

During the presented valve cycle, the transvalvular venous flow was approximately plug-shaped, as shown in Fig 8A. The slight asymmetrical flow patterns at different times indicated that the flow orientation had small oscillations with the leaflets oscillating.

According to the *in vitro* experiment of Qui et al. [32] that velocity was almost uniformly distributed normal to the longitudinal cross-section, the volumetric flow rate *Q* ($Q=\pi [(d_{0}+2h_{s}^{\prime })\int_{0}^{l_{GOA}}v^{f}dy]/4$) between the leaflets was integrated. As shown in Fig 8B, the peak (transvalvular) flow rate *Q* was 11.68 cm^3^/s at 0.28 s, while the peak (transvalvular) velocity was 40.5 cm/s. The mean maximum velocity was 28.89 cm/s for the vein diameter 0.691 cm, which complied with the linear inverse relationship between mean maximal diameter and mean peak venous velocity reported in [50]. Further, the reverse flow started at 0.50 s at the outlet and the peak reverse flow velocity appeared around the orifice and was –4.6 cm/s (Fig 6C), which was consistent with the findings of Lurie et al. [51] that the normal valve could be closed when the reverse flow velocity was low.

**Fig 9:**
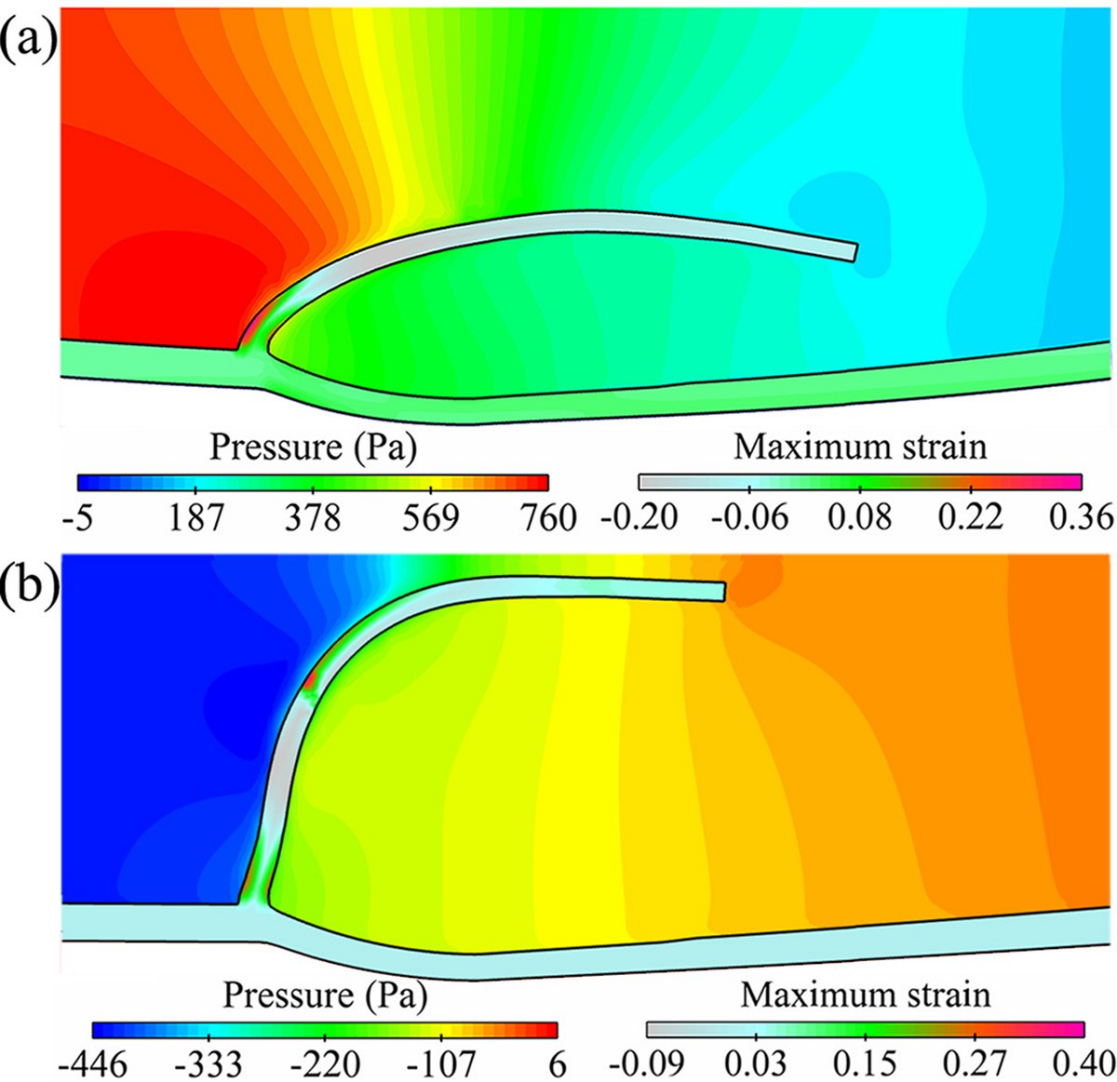
Contours of the pressure and the first principal strain (MPSN) at the maximum opening orifice and the closed valve configuration.

Pressure drop in the vein is another feature of the valve functioning. As shown in Fig 9, the pressure difference was the greatest at the leaflet base (hinge) and smallest at the tip. When the opening orifice was maximum, the pressure gradient was 220 Pa (1.67 mm Hg) and less than 3 mm Hg, agreeing with the stipulation for a biological venous prosthesis in opening function [11,14,52]. Accordingly, the first principal strain on the anterior surface of the valve base was the greatest of the vein. The negative strain appeared on the upper surface of the valve, indicating the compressive loading from the ejected flow. In the closed phase, the pressure gradient reversed its direction, and a significant strain occurred on the upper surface owing to the flexure of the leaflet in the upstream direction.

**Fig 10:**
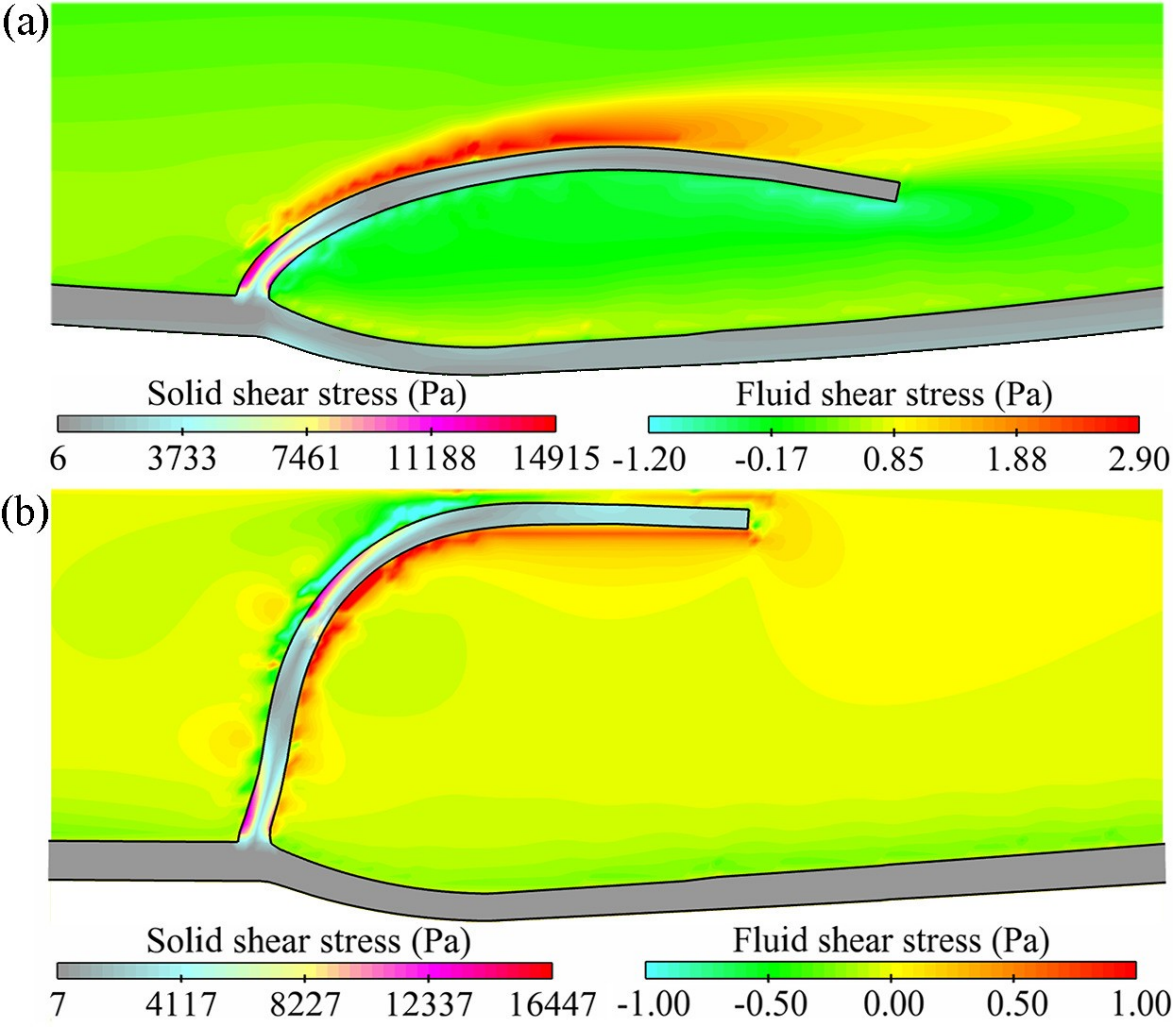
WSSs of blood and valve. (a) In the maximum opening state, 0.30 s. (b) In the closed state, 1.00 s.

The WSSs of blood and valve are illustrated in Fig 10 to verify the mechanotransduction further against the existing calculated results. As the blood outside the wall was assumed as the background fluid for immersing the vein, its stress is not given here. During the opening phase, evident fluid shear stress (2.8–3.6 Pa) appeared on the upper surface of the leaflet, which was maximum and close to 3.3 Pa as calculated by Soifer et al. [19]. The maximum solid shear stress in the opening phase occurred on the base region, and the resulting stress was between 5.0 kPa [19] and 20.0 kPa as reported by Chen et al. [33] and Ariane et al. [20]. In the closed phase, the fluid wall shear stress (FWSS) exhibited apparent decrease while the solid wall shear stress (SWSS) increased, which was owing to the coaptation of the leaflet. The low FWSS in the sinus pocket region decreased from the maximum value of 0.8 Pa to 0.08 Pa. It suggested that the sensitive region was of significance in influencing endothelial mechanism and promoting thrombosis or intimal hyperplasia, being consistent with the biological data available [53].

Note that a brief parameter analysis is further presented in S2B Appendix and S2 Fig for the length of the leaflet tip and the contact parameters.

### Effect of valve lesion on valve closure

**Fig 11:**
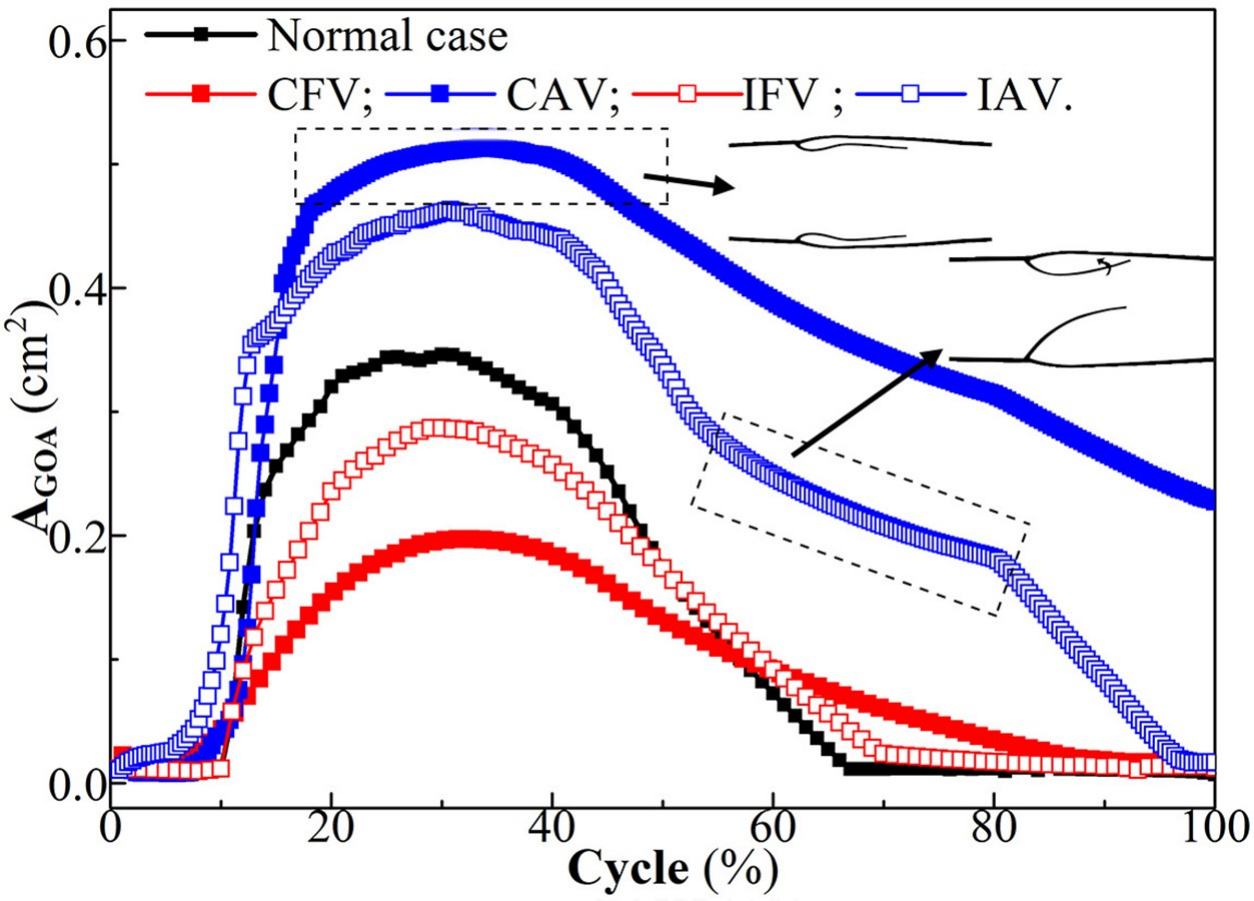
Time variation of the GOA.

The first focus of this simulation was to determine the extent of effects of valve lesions on the valve closure. As illustrated in Fig 11, the GOA was inversely proportional to the structural stiffness of the valve. In the equilibrium phase, A_GOA_(CAV) > A_GOA_(IAV) > A_GOA_(Normal) > A_GOA_(IFV) > A_GOA_(CFV).

**Fig 12:**
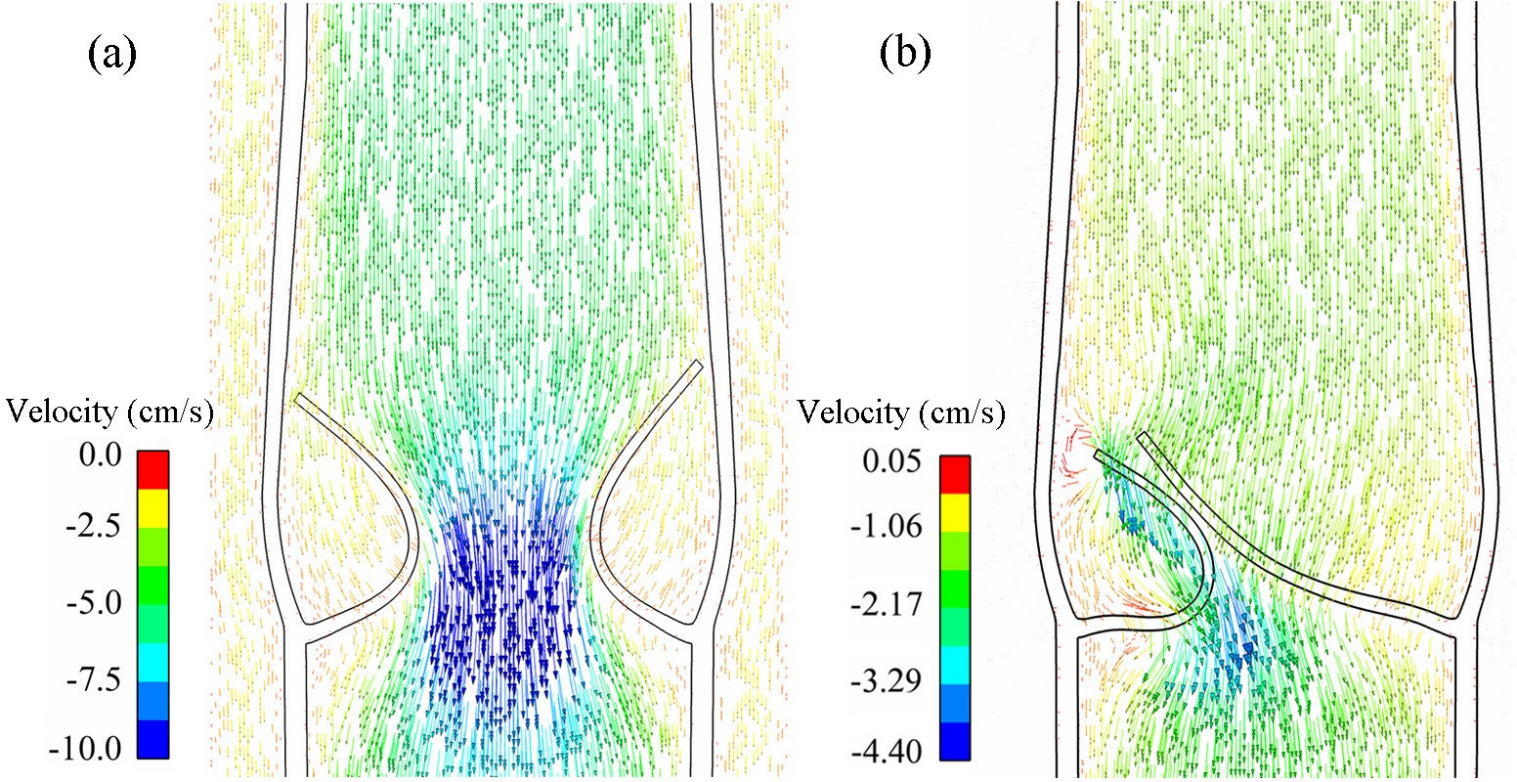
Valve configuration and flow streamlines in the cases of (a) CAV and (b) IAV at 1.00 s.

Furthermore, the duration of each phase in the proposed cycle was altered due to different vein responses. Especially for the CAV, the valve remained incompletely closed at 1.0 s, as shown in Fig 12A. Each leaflet finally leaned against the sinus, with rotations of 56.09 degree (the No. 1 leaflet) and 44.9 degree (the No. 2 leaflet) and repulsion forces of 0.32 dyne/cm (No. 1) and 1.80 dyne/cm (No. 2). Similar to the leaflet of the CAV, the No. 1 leaflet (atrophic) of the IAV leaned on the sinus (Fig 12B), and the No. 2 leaflet (healthy) rotated anticlockwise 74.9 degree to close the valve. It seemed like a mono-leaflet valve [33]; the healthy leaflet had the sole role in presenting the reverse flow. Except the GOA, only small variations in durations of the equilibrium phase were found in the fibrotic cases relative to the normal valve.

### Effect of valve lesion on venous volume

**Fig 13:**
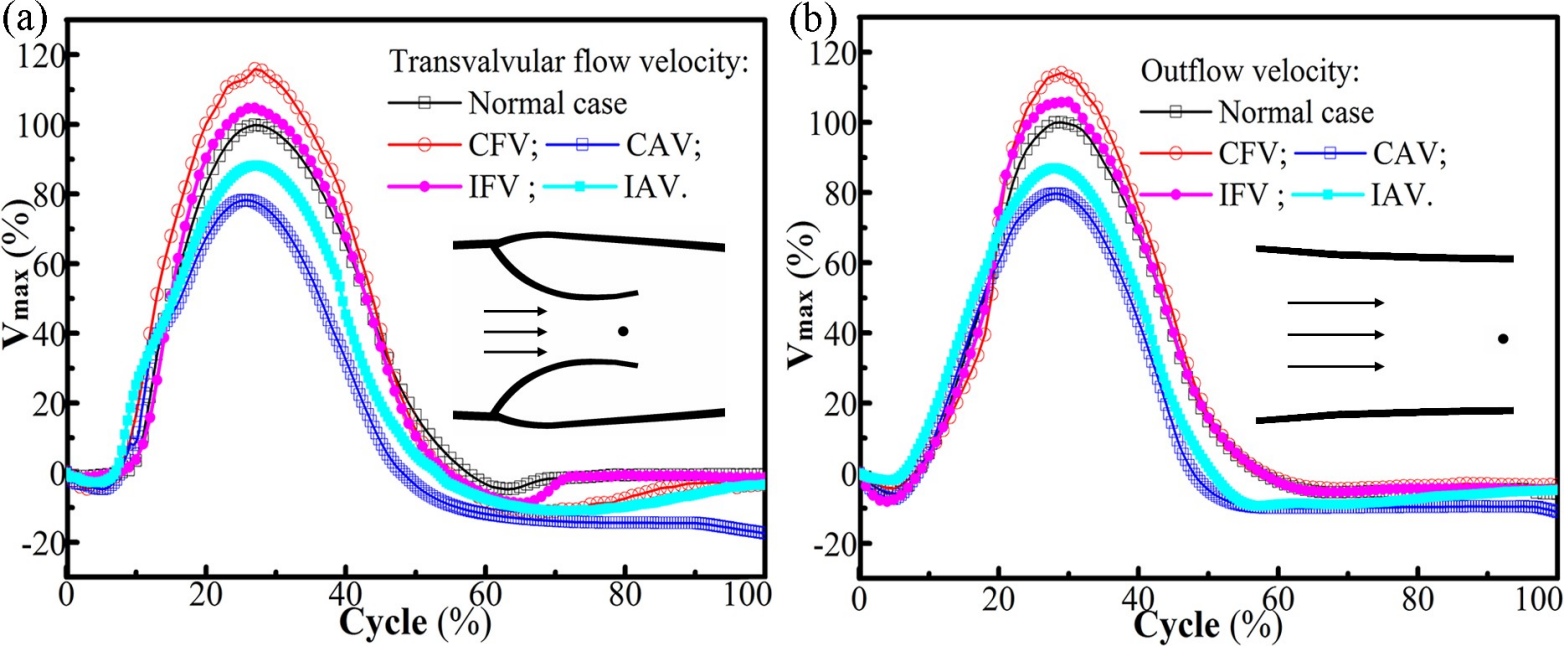
**Normalized maximum velocity of (a) the transvalvular flow and (b) the outflow.** The maximum velocities for the normal valve are calibrated to 100%.

The variations of valve closure induced by the valve lesions led to particular flow patterns in the vein. Although the flows became more complicated, the transvalvular flow velocity and outflow velocity were almost proportional to the structural stiffness of the valve. The flow characteristics of the atrophic cases are shown in Fig 13. For fibrotic cases, smaller motion range resulting from the less elasticity caused a stronger jet and vice versa, which was similar to that reported by Soifer [19].

The influence on the venous volume [54] could be quantitatively found. Using the time integration, the venous volumes of the antegrade and retrograde flows were determined (Table 5). For one cycle, the forward flow rates were related to the valve lesions as follows: *Q*(CAV) > *Q*(IAV) > *Q*(Normal) > *Q*(IFV) > *Q*(CFV). Instead, the law of the reverse flow rate was unclear. Particularly for the atrophic lesion, the venous volume of the reverse flow still increased at the end of the proposed cycle because the valve leaflets failed to close. Such a situation remained even after the proposed cycle (not presented in this study) and the duration exceeded 0.5 s, which is a marker of the so-called “reflux” in physiology [55]. Also, the reversed venous volume in the CAV led to an evident decrease in its transporting capacity.

**Table 5:** Venous volume and the related time of different valves for one cycle.

Valve	Forward flow (cm^3^)	Reverse flow (cm^3^)	Critical time (cycle)
**Normal**	3.50	−0.030	56%
**CFV**	1.98	−0.064	53%
**CAV**	2.97	−1.150	45%
**IFV**	2.89	−0.040	55%
**IAV**	3.20	−0.241	56%

### Effect of valve lesion on WSS

**Fig 14:**
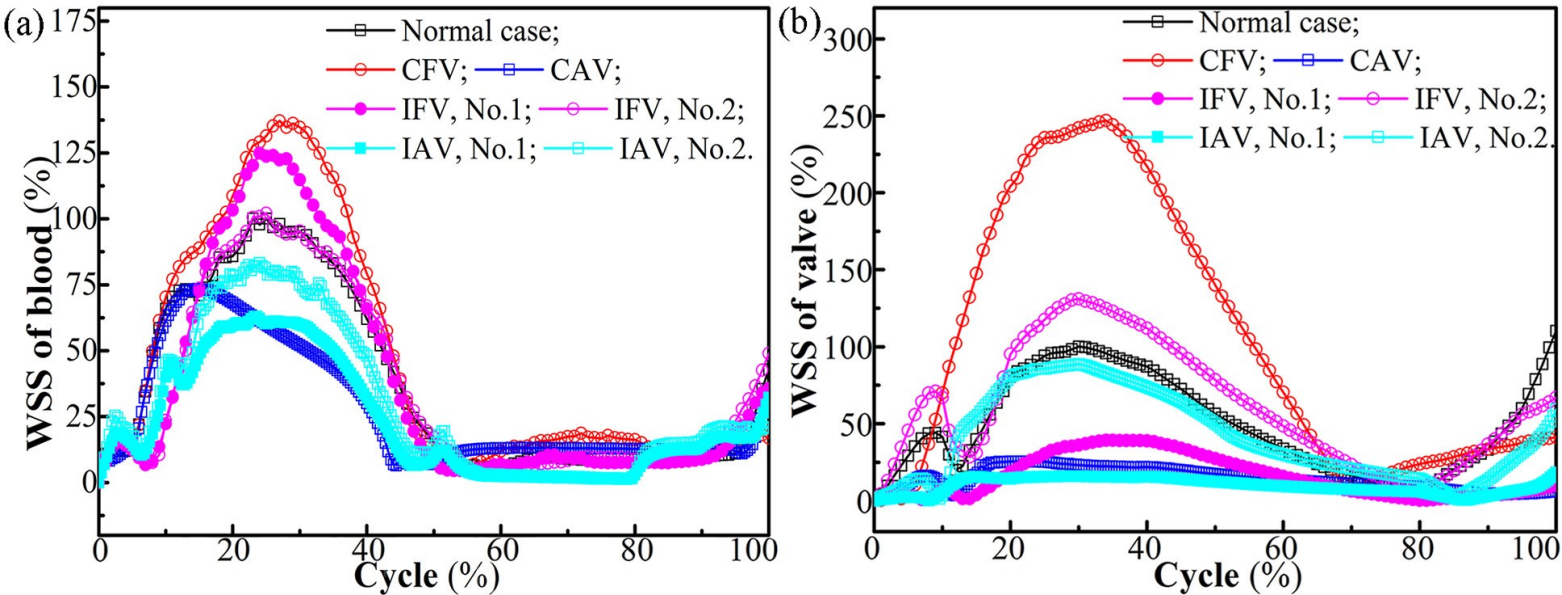
Maximum WSS of (a) the blood and (b) the leaflet for different cases.

As illustrated in Fig 14A, the maximum blood WSS of different cases differed proportionally to the structural stiffness of the valve during the opening phase, given its relationship with the orifice area and blood velocity. The maximum leaflet WSS seemed to follow the similar law, as shown in Fig 14B. Both the FWSS and the SWSS of the CFV were the highest, and those of the CAV were the smallest.

**Fig 15:**
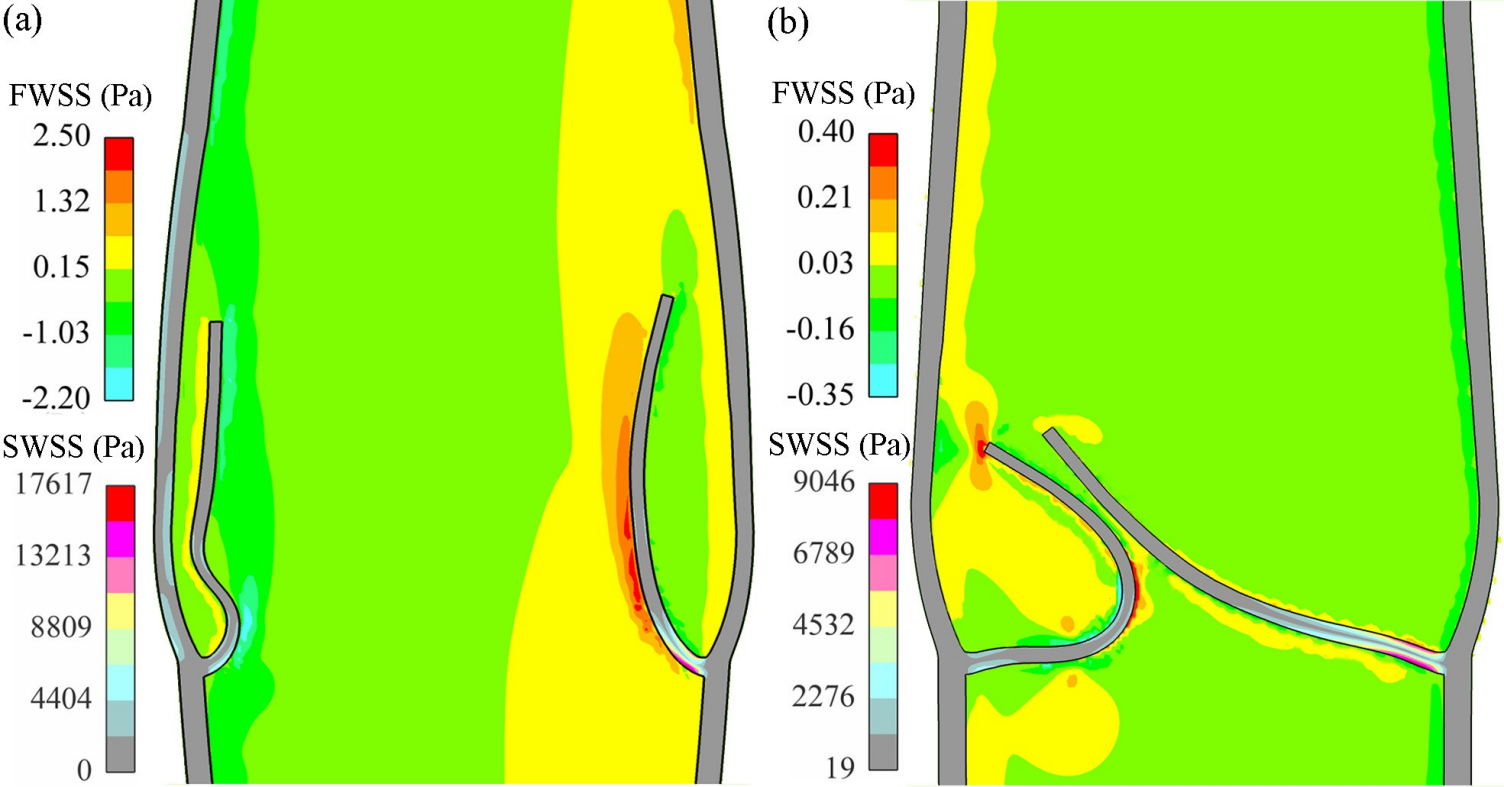
Blood and leaflet WSSs (a) at 0.30s and (b) at 1.0 s for the IAV.

For the atrophic leaflet, it was further seen that the FWSS and SWSS were subjected to a peak in the early opening phase, which should be related to its weak resistance to the inflowing blood pressure, as shown in Fig 15A. In the closed phase, a second increase in the SWSS was seen for all the cases, which was consistent with that found in another numerical study of the pathological venous leaflet [19]. This trend needs further exploration. For example, the No. 2 leaflet (healthy) of the IAV had a second increase in its WSS, which was induced by the tension when the No.2 leaflet rotated toward the No. 1 leaflet, as shown in Fig 15B.

**Fig 16:**
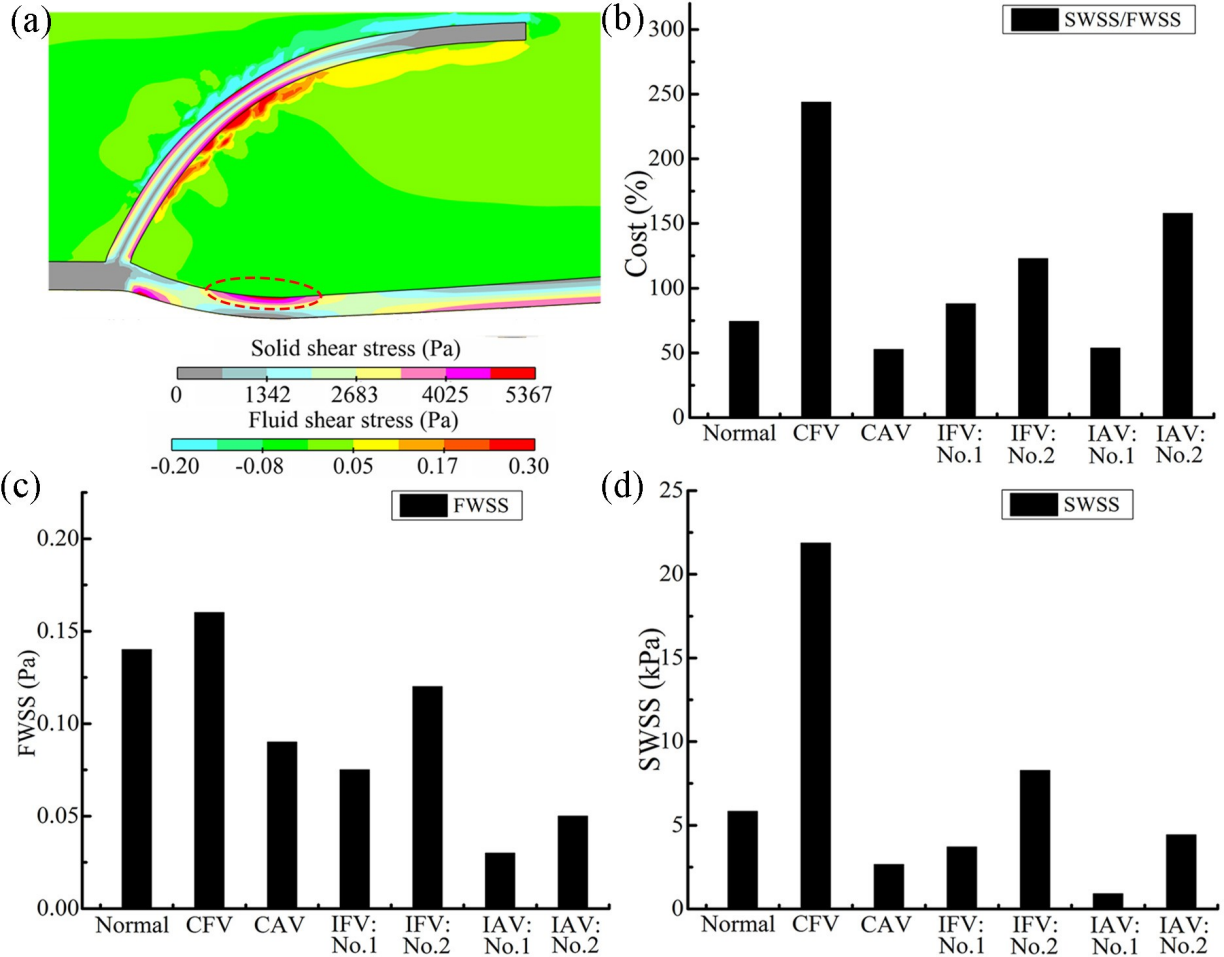
(a) Blood and leaflet WSSs in the closed phase for the CFV. (b) Mechanical cost of the valve for different cases. (c) SWSS and (d) FWSS at the leaflet backside.

The primary mechanism of valve lesions influencing the valve biology was that the low-shear-stress regions in the pockets behind the leaflets might cause flow stagnation and enhance destruction of the vein. Both the FWSS and SWSS were analyzed for the base region of the leaflet because the sinus side of the region was critically sensitive to mechanical stimuli [56]. In the CFV, an apparent shear stress was seen on the sinus, where the endothelial cells existed, indicating significant mechanical stimuli, as shown in Fig 16A. Another apparent stress occurred on the exterior sinus wall owing to the material gradient between the wall and the sinus. (Note that the sinus dynamics are not detailed to focus on the valve in this study.)

As illustrated in Fig 16B, low blood WSS in the stagnation and high valve WSS in the base region were identified through a mechanical cost (mechanical cost = solid WSS/fluid WSS) [33]. Both FWSS and SWSS at the backside of the sinus were used, as shown in Fig 16C and 16D. The moment was chosen when the FWSS was the lowest and the decreasing SWSS was still high, such as 0.54 s for the CFV (Fig 16A). The apparent difference in the cost was normalized as 1:3.28:1.66:2.13 for the leaflet of the normal valve, the leaflet of the CFV, the healthy leaflet of the IFV, and the healthy leaflet of the IAV. It showed that the biomechanical conditions became hemodynamically less favorable in the CFV [56].

## Discussion

This study presented a 2D numerical venous model to mimic the realistic valve cycle. It analyzed the valve dynamics and hemodynamics of the pathological situation in veins, such as venous incompetence and blood reflux. Limited by the scanty information and adequate attention, previous relevant studies were inadequate, especially most of them focusing on the healthy leaflets. In venous valve modeling, the wall and sinus were fixed [12,13] and the transporting capacity (venous volume) was seldom mentioned [20]. A modified IFEM modeling was employed on the basis of the anatomical data [17,24,57] and the physiological pressure conditions [4] to gain a new understanding of the valve cycle in the diseased vein. The sensitivity analyses of mesh size and time step for the modified IFEM were verified using small models before the valve modeling.

The proposed 2D model represented the longitudinal cross-section of a 3D geometry. The velocity and pressure fields were comparable and still available for biomechanical analysis [29,30], although the examined parameters in the present model would probably differ from 3D valve model in results, such as the vortical and helical flows. A 2D model has a distinct advantage of less cost on finite element computation, which lowers the dimensions of matrix storage in computational modules and accelerates the solution time with limited computing power. It took 23.52 h on the available hardware to solve one case, while similar models in 3D could take weeks or more.

The benchmark problem of a normal venous valve cycle was validated by comparing the dynamics and hemodynamics against the published ones. Although some detailed characteristics of the flow were not obvious owing to the rough assumption of the sinus structure and material, the classical four phases proposed by Lurie et al. [8] were observed in a single cycle. In the opening phase, the transvalvular pressure gradient decreased from the leaflet base to the tip and was lower than that stipulated in the biological venous prosthesis [52], indicating that the modeling valve was soft and its opening function was closer to that of the native valve. Also, the maximum opening orifice was within the physiological level reported by Lurie et al. [48]. Although the specific extent of the sinus distension contributing to the opening orifice was seldom mentioned in *in vitro*/*in vivo* studies [24], the present study suggested that the compliance of the venous tissues had a known positive role in the valve opening. The significant role of the flexural behavior of the elastic leaflet [58] in the valve cycle was also detected because the valve could be closed under the low reverse flow velocity and neutral pressure gradient [51]. Particularly, the leaflet stress had a second increase when the leaflet became closed. The low-fluid WSS in the sinus pocket region and high-solid WSS in the leaflet base region complied with the common clinical knowledge. Their agreement with the existing numerical results suggested the feasibility of the stress analysis in the present valve modeling.

The leaflet opening was inversely proportional to the venous structural stiffness owing to the valve lesion induced by the varying elasticity. The atrophic leaflet particularly failed to be closed or was so-called incompetent. The atrophic leaflet leaning on the sinus wall might be a potential sign of abnormal valve cycle because Lurie et al. [8] reported that the valve leaflets did not open all the way out to touch the sinus wall but made an angle of about 30 degrees with the wall. In the equilibrium phase, the posterior surface of the leaflet attached on the sinus wall with a small gap (S3A Fig). Similar to the CAV (S3B Fig), the atrophic leaflet in the IAV displayed extremely weak resistance to the hemodynamic action. The IAV could be reluctantly closed owing to the large rotation of the healthy leaflet. It showed that the healthy leaflet had a dominant role in valve functioning and was subjected to relatively higher FWSS and SWSS. Therefore, the healthy leaflet in IAV was prone to destruction and a commissural reflux canal [9] might even be shaped after a long-term performance. Hence, the atrophy lesion might be considered a significant risk factor leading to incompetence and reflux. This valve lesion gained adequate attention from researchers because the prevalence of incompetent valves (in the healthy internal jugular vein) was 36.8%–38.4% [5], while 28.9% of venous reflux finally developed into varicose veins [55]. The described mechanism might help in elucidating the relationship between the incompetent valve and the reflux, providing a prospective understanding of the valve closure mechanism in the diseased vein with the valve lesions.

Valve fibrosis due to phlebitis was another significant factor in venous insufficiency. Fibrotic leaflets were characterized by increased collagen and reduced elastin content, and thus became rigid. Then, the decreased motion range of fibrotic leaflet led to a stronger jet. Moreover, the transvalvular blood volume became smaller, which could be supposedly regarded as a signal of a decrease in blood transporting capacity. Although such a shift in volume could be accommodated within the large venous system, severe symptoms (such as blood pooling) usually developed from the local worsening of the transporting capacity [4]. The described mechanism might provide a preliminary understanding of the hemodynamics of the diseased vein with fibrosis. Also, the similar flow feature could be exactly employed as a pathological signal for the fibrotic lesion.

The hemodynamics and valve dynamics are related to the primary mechanism of valve biology. In this study, low-blood WSS was seen for all the cases on the sinus pocket. The base region of the valve experienced the highest leaflet stress, thus accelerating the negative influence on the biology because blood and leaflet WSSs of an abnormal valve are altered in the opposite trend simultaneously, such as in fibrosis. Suppose that the biomechanical condition was in an equilibrium, the alterations of the WSSs induced by the valve lesion would interrupt the equilibrium because FWSS was easier to be sensed by the endothelial cells. Then, the mechanotransduction would initiate, and thrombotic and inflammatory cells in sinus regions might accumulate and adhere [59]. The results of cost function indicated a signal of the deterioration of the pathological situation because the lower cost function correlated with less intimal hyperplasia. The CFV might be the undesirable case biologically because subsequent damage of the fibers could lead to the deterioration of the pathological situation [60]. This described mechanism was really significant. It could be employed to provide quantitative proof for early detection of the deterioration symptoms in the diseased vein.

Additionally, this novel study used an immersed type method and an adhesive contact method in exploring the pathological valve. It is still a preliminary study, although it provided a valuable understanding of the abnormal behavior in the vein, such as incompetency, venous reflux, obstruction and closure. The geometry and constitution still need improvement. For example, the sinus feature is unclear and its material property is assumed between those of the wall and the leaflet. Thus, the employed bending stiffness of the leaflet resulted in a conservative valve configuration in the closing-closed phase. Also, the assumed gap between the leaflets was inevitably introduced owing to the limited choice of the present contact methods. A comparison of the present modeling with the physiological venous valve still showed limitations and room for improvement, whereas the agreement with the existing results suggested that the modeling was sufficiently capable of simulating the venous valve cycle. According to the described mechanisms in the diseased vein in this study, the influence of the presented four valve lesions was clearly known. Also, the corresponding biological mechanisms need to be revealed so that the understanding of the results can be improved. This may further assist in achieving complete understanding, efficient bioprosthesis, and more effective treatment of the diseased valve.

## Conclusions

A fluid–structure interaction model was constructed using a modified IFEM in this study to mimic the venous valve cycle. The contact action between venous tissues was explored using the adhesive contact algorithm. The presented model provided a robust and high-fidelity solution by comparing with the established model using the FSI method. The resulting valve dynamics and hemodynamics were also validated by the existing results. This novel study quantitatively and qualitatively analyzed venous incompetence and blood regurgitation in a pathological valve. This simulation provided a prospective understanding of the effect of valve lesion on the venous valve cycle. Comparison between the healthy valve and the pathological valve further reflected the relationship between the valve lesion and the valve functioning. The presented results provided the understanding of the mechanisms of venous incompetency and reflux in the diseased valve. The preliminary understanding of the relationship between healthy and unhealthy leaflets in the incomplete lesion was also achieved.

The present study was still in its preliminary stages and had some limitations, although the valve modeling was capable of simulating a normal valve cycle and providing the valve dynamic and hemodynamic results. Further studies should focus on improvements, such as 3D modeling, the realistic closed valve configuration, and the helical flow. The present geometry and constitution should also be improved to get closer to the native venous valve structure and function. Further investigation of venous valve lesion is required, particularly *in vivo*/*in vitro*, to improve the current understanding for an efficient bioprosthesis and more effective treatment of the diseased valve.

## Supporting information

S1 Fig Sensitivities of the mesh size and time step. (a) Material and geometry of test model. (b) Effect of mesh size on solid deformation. (c) Effect of time step size on solid deformation. (d) Fluid velocity of Point Q with different mesh sizes. (e) Fluid velocity of Point Q at different time step sizes.
(TIF)

S2 Fig Sensitivities of the geometry and contact factors. (a) Geometric change in the leaflet length. (b) Contact force on the leaflets. (c) Sensitivities of the GOA to ***d***_***v***_ and *ε*. (d) Sensitivities of the contact force to ***d***_***v***_ and ***ε***. Here A is the result of the normal valve, B, the result of the larger contact stiffness and C the result of the shorter leaflet.
(TIF)

S3 Fig Flow patterns and valve configurations of (a) completely atrophic valve and (b) incompletely atrophic valve in the equilibrium phase.
(TIF)

S1 Appendix Second order fractional step finite element method (SOFES-FEM). (DOCX)

S2 Appendix *A*, Sensitivity of the mesh density and time step in the proposed modified IFEM. *B*, Factor analyses of geometry and contact parameters.
(DOCX)

S1 Movie Blood and valve kinematics for the normal. (AVI)

S2 Movie Blood and valve kinematics for the CFV. (AVI)

S3 Movie Blood and valve kinematics for the CAV. (AVI)

S4 Movie Blood and valve kinematics for the IFV. (AVI)

S5 Movie Blood and valve kinematics for the IAV. (AVI)

S6 Movie Blood and valve dynamics for the CAV. (AVI)

S7 Movie Blood and valve dynamics for the IAV. (AVI)
