# Supplementary figures and images for: Effect of valve lesion on venous valve cycle: A modified immersed finite element modeling
Source: PLoS One. 2019 Mar 4;14(3):e0213012. doi: 10.1371/journal.pone.0213012 (PMC6398833; doi:10.1371/journal.pone.0213012)

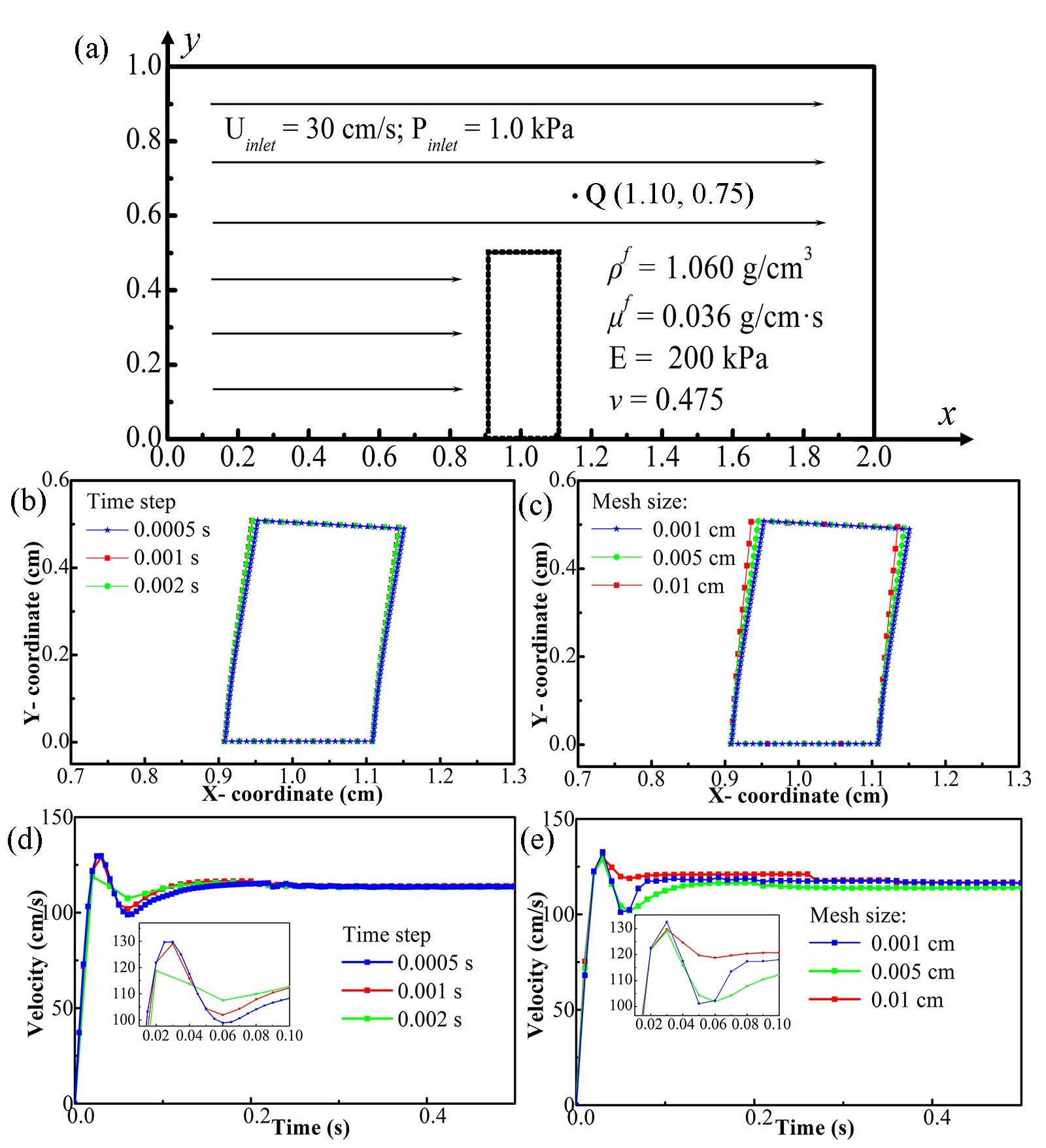

Supplement: S1 Fig — (a) Material and geometry of test model. (b) Effect of mesh size on solid deformation. (c) Effect of time step size on solid deformation. (d) Fluid velocity of Point Q with different mesh sizes. (e) Fluid velocity of Point Q at different time step sizes. (TIF) [file pone.0213012.s001.tif]

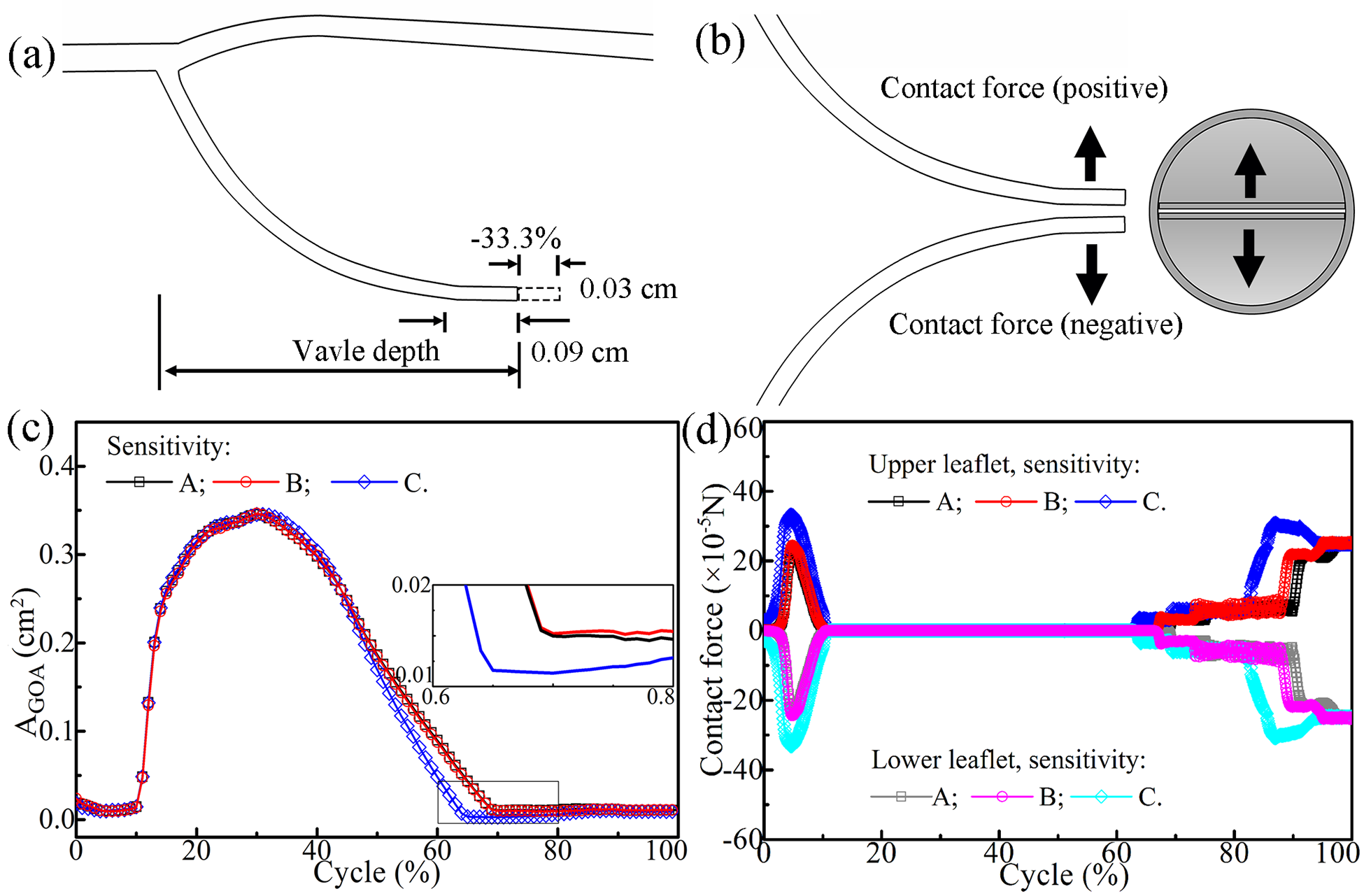

Supplement: S2 Fig — (a) Geometric change in the leaflet length. (b) Contact force on the leaflets. (c) Sensitivities of the GOA to dv and ε. (d) Sensitivities of the contact force to dv and ε. Here A is the result of the normal valve, B, the result of the larger contact stiffness and C the result of the shorter leaflet. (TIF) [file pone.0213012.s002.tif]

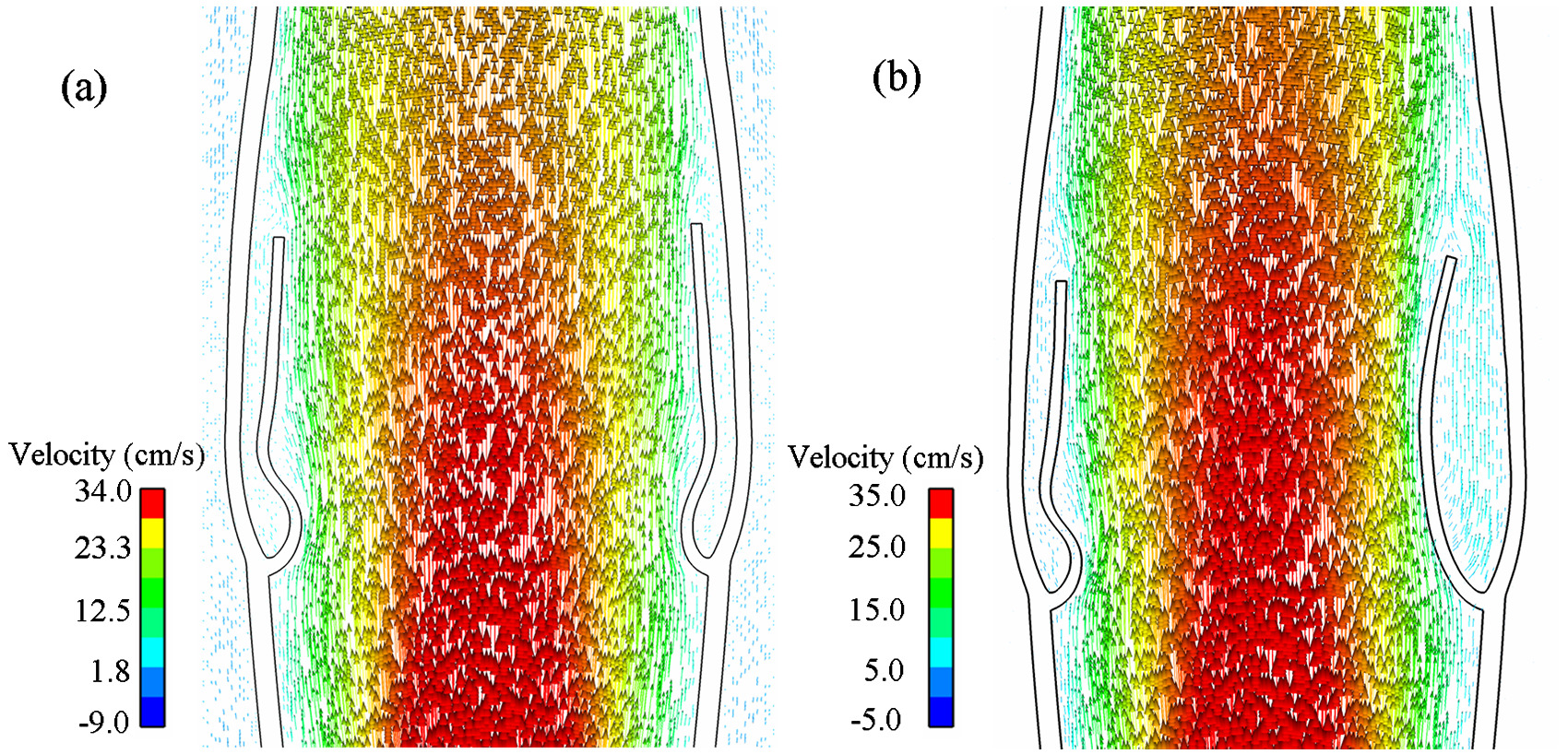

Supplement: S3 Fig — Flow patterns and valve configurations of (a) completely atrophic valve and (b) incompletely atrophic valve in the equilibrium phase. (TIF) [file pone.0213012.s003.tif]
