# Supplementary material for: Effect of valve lesion on venous valve cycle: A modified immersed finite element modeling
Source: PLoS One. 2019 Mar 4;14(3):e0213012. doi: 10.1371/journal.pone.0213012 (PMC6398833; doi:10.1371/journal.pone.0213012)
Supplement: S1 Appendix — (DOCX) [file pone.0213012.s004.docx]

**S1 Appendix**

In this section, we provide a brief introduction to the techniques employed in the modified IFEM.

*A.* *Second order fractional step finite element method (SOFES-FEM)*

A second order fractional step finite element method [1] was used to improve the pressure stability in the N-S equations. The method included two sub-steps. Assuming that $t$ was the previous time and $\Delta t$ was time step size, then the current time was labelled as $t+\Delta t$. In the first sub-step, an intermediate velocity was expressed as follows:

${}^{t+\xi\Delta t}{\hat{v}_{i}}=\xi{}^{t+\Delta t}{v_{i}^{*}}+\left( 1-\xi\right){}^{t}{v_{i}}$ (A)

at the intermediate time $t+\xi\Delta t$, where $\xi$ is a constant. Then, the current velocity ${}^{t+\Delta t}{v_{i}^{*}}$ was obtained by solving:

$\frac{\rho}{\Delta t}\left( {}^{t+\Delta t}{\hat{v}_{i}}-{}^{t}{\hat{v}_{i}} \right)+\rho({}^{t+\xi\Delta t}{\hat{v}_{i,j}})({}^{t+\xi\Delta t}{\hat{v}_{i}})=-{}^{t}{\hat{p}_{,i}}+\mu({}^{t+\xi\Delta t}{\hat{v}_{i,j}}+{}^{t+\xi\Delta t}{\hat{v}_{j,i}})+\rho f_{i}$

(B)

In the second sub-step, the pressure correction equation

$\nabla_{\boldsymbol{x}}{}^{t+\Delta t}{p^{*}}=\eta{}^{t}p-\rho\left( {}^{t+\Delta t}{\boldsymbol{v}^{*}}-{}^{t+\Delta t}{\hat{\boldsymbol{v}}} \right)/\Delta t$ (C)

was used to calculate the current pressure ${}^{t+\Delta t}{p^{*}}$ with another constant $\eta$. Corresponding to the second-order Crank–Nicolson scheme [2], $\xi$ = 0.5 and $\eta$ = 0.5 were chosen.

**References**

1. Codina R. Pressure stability in fractional step finite element methods for incompressible flows. J Comput Phys. 2001;170: 112–140. doi:10.1006/jcph.2001.6725

2. Heywood JG, Rannacher R. Finite-Element Approximation of the Nonstationary Navier-Stokes Problem Part IV : Error Analysis for Second-Order Time Discretization. SIAM J Numer Anal. 2016;27: 353–384. doi:doi.org/10.1137/0727022
