# Supplementary material for: Effect of valve lesion on venous valve cycle: A modified immersed finite element modeling
Source: PLoS One. 2019 Mar 4;14(3):e0213012. doi: 10.1371/journal.pone.0213012 (PMC6398833; doi:10.1371/journal.pone.0213012)
Supplement: S2 Appendix — A, Sensitivity of the mesh density and time step in the proposed modified IFEM. B, Factor analyses of geometry and contact parameters. (DOCX) [file pone.0213012.s005.docx]

**S2 Appendix**

In this section, we firstly provide a sensitivity analysis of mesh density and time step to the immersed finite-element-modelling [1], then anther brief parameter analyses of geometrical dimension and the contact force [2] are given.

*A.* *Sensitivity of the mesh density and time step* *in the proposed modified IFEM*

Because the presented interpolation between the fluid and solid is a weak coupling, the fluid solver is subject to Courant–Friedrichs–Lewy (CFL) type time-step constraint, where $CFL=\frac{v\Delta t}{h_{e}}$ [3]. The mesh size $h_{e}$ is the other constraint. The sensitivity analyses of the time step size $\Delta t$ and the mesh size $h_{e}$ were performed by a heuristic assessment. A flow configuration consisting of an elastically mounted unilateral leaflet in a uniform flow was chosen. Geometry, property parameters and boundary conditions of the model were given in Figure A in Fig S1. This canonical problem shared typical FSI features of valve cycle, while the simple configuration made it amenable to analysis. Choosing quadrilateral fluid mesh of 5000, all the time step sizes (0.0005 s, 0.001 s and 0.002 s) converged to the same solution, as shown in Figures B and D in S1 Fig. By comparing the resulted solid configurations, Figures C and D in S1 Fig show that little accuracy could be improved when mesh resolution was increased into a certain extent (regular quadrilateral fluid mesh of 800, 5,000, and 20,000 elements, respectively).

*B.* *Factor analyses of geometry and contact parameters*

To further validate the computational model, effects of geometry and contact factors are discussed, as shown in S2 Fig. We chose $d_{v}$ and $\epsilon$ as influence factors. Their sensitivities were analyzed quantitatively through the valvular deformation and the contact force. In geometry, the leaflet apex was specially designed to be flat so as to consider the effect of the leaflet length, which was then shortened by 33.3% (see Figure A in S2 Fig). In the contact module [2], $\epsilon$ was $-$40.0 J by enlarging the contact stiffness ten times. Figure C in S2 Fig illustrates that shorter leaflet resulted in early valve closure. The shorter leaflets were sliding when closing since the friction became smaller with limited contact surfaces. Figure D in S2 Fig shows that higher $\epsilon$ led to distinctly larger repulsion at the initial time. In the following phase, $\epsilon$ affected it little. The final repulsion forces in the presented three cases converged to 2.6$\times$10^-4^ N.

**References**

1. Zhang L, Gerstenberger A, Wang X, Liu WK. Immersed finite element method. Comput Methods Appl Mech Eng. 2004;193: 2051–2067. doi:10.1016/j.cma.2003.12.044

2. Fan H, Ren B, Li S. An adhesive contact mechanics formulation based on atomistically induced surface traction. J Comput Phys. 2015;302: 420–438. doi:10.1016/j.jcp.2015.08.035

3. TAKIZAWA K, TEZDUYAR TE. Space-time fluid-structure interaction methods. Math Model Methods Appl Sci. 2012;22: 1230001. doi:10.1142/S0218202512300013
